# DNA Fingerprinting of Chinese Melon Provides Evidentiary Support of Seed Quality Appraisal

**DOI:** 10.1371/journal.pone.0052431

**Published:** 2012-12-20

**Authors:** Peng Gao, Hongyan Ma, Feishi Luan, Haibin Song

**Affiliations:** College of Horticulture, Northeast Agricultural University, Harbin, Heilongjiang Province, People’s Republic of China; Kansas State University, United States of America

## Abstract

Melon, *Cucumis melo* L. is an important vegetable crop worldwide. At present, there are phenomena of homonyms and synonyms present in the melon seed markets of China, which could cause variety authenticity issues influencing the process of melon breeding, production, marketing and other aspects. Molecular markers, especially microsatellites or simple sequence repeats (SSRs) are playing increasingly important roles for cultivar identification. The aim of this study was to construct a DNA fingerprinting database of major melon cultivars, which could provide a possibility for the establishment of a technical standard system for purity and authenticity identification of melon seeds. In this study, to develop the core set SSR markers, 470 polymorphic SSRs were selected as the candidate markers from 1219 SSRs using 20 representative melon varieties (lines). Eighteen SSR markers, evenly distributed across the genome and with the highest contents of polymorphism information (PIC) were identified as the core marker set for melon DNA fingerprinting analysis. Fingerprint codes for 471 melon varieties (lines) were established. There were 51 materials which were classified into17 groups based on sharing the same fingerprint code, while field traits survey results showed that these plants in the same group were synonyms because of the same or similar field characters. Furthermore, DNA fingerprinting quick response (QR) codes of 471 melon varieties (lines) were constructed. Due to its fast readability and large storage capacity, QR coding melon DNA fingerprinting is in favor of read convenience and commercial applications.

## Introduction

Melon (*C. melo* L., 2n = 24), which belongs to *Cucumis*, Cucurbitaceae, is an important vegetable crop worldwide. Among Cucurbitaceae, *C. melo* is one of the most important cultivated cucurbits, due to its economic and nutraceutical importance [1].

Global melon yield in 2009 was over 25.5 million tons, and in the past 30 years has increased 1.7 fold with an average annual growth rate of 3.6%. China is the biggest melon producer in the world, accounting for about half of global production in recent years. The total yield in China in 2009 was over 12.22 million tons (FAO Statistics 2009, http://faostat3.fao.org/home/index.html#VISUALIZE_BY_DOMAIN).

In China, a large number of melon varieties are being released and commercialized every year. While some of these commercial varieties have the same parents, many different trade names (synonym) are used, and on the other hand, some of the commercial varieties have the same names but their parents are different (homonym). This situation in China is widespread, which confuses melon breeders, seed producers, as well as melon producers, and damages their economic interests, including also negative influence on exporting of seeds and fruit produce to other countries due to the confusion and inconvenience it has caused. Another situation is that some seed companies’ lax quality control results in problems of seed purity and affects the interests of farmers seriously. On the other hand, assessment of genetic variation is important for melon breeding: efficient management, protection and application of germplasm resources [2]. Detection and utilization of the genetic variation and cultivar identification are thus some important tasks for melon breeders.

Traditionally, evaluation of seed quality and germplasm management has been based on morphological descriptors. However, many modern varieties and hybrids are phenotypically less distinct than traditional varieties, and morphological evaluation is more difficult. Furthermore, identification based on morphological descriptors is time consuming and often influenced by environmental conditions. All of these factors increase the difficulties of monitoring the melon seed market. Therefore, new, reliable, and timesaving methods are needed for accurately assessing cultivar identification and genetic diversity [3].

Molecular marker technologies offer alternatives for the identification of cultivars and genetic diversity. Techniques have included, for melon, the utilization of isozymes, random amplified polymorphic DNA, amplified fragment length polymorphisms, single nucleotide polymorphisms, and simple sequence repeats (SSRs) [4]–[7]. Among all the marker systems, single nucleotide polymorphisms are the best for marker-based cultivar identification and genetic diversity studies [6]. However, the high cost and the difficulties associated with single nucleotide polymorphism genotyping prevent it from being used in most melon breeding programs [8], and especially by monitoring laboratories supported by local governments and small breeding laboratories in developing countries such as China.

SSRs are a viable alternative for these laboratories, because the co-dominant and multi-allelic nature and high reproducibility of SSR markers are sufficient for identification of germplasm and genetic variation [9]–[11]. More importantly, the experimental conditions necessary to perform SSR genotyping are realizable for small laboratories. During the past decade, much data have been generated using SSR markers for the evaluation of the genetic diversity of crops such as barley, wheat, sorghum, potato, rice, and maize [12]–[21]. An overview of these results indicates that the SSR marker system is a powerful and economical tool for analyzing crop genetic diversity.

In melon, SSR markers have been used broadly to define genetic associations among botanical groups and commercial market classes [22]–[25], and assessments of the major primary and secondary geographic origins of melon diversity. However, there has been very few polymorphism analysis of Chinese marketable melons. Therefore, it is necessary to establish an SSR-based DNA fingerprint database for melon which could provide support for evaluations of varietal purity and authenticity.

In the present study we developed and validated a core set of SSR markers to establish a DNA fingerprint database for melon. We detected polymorphism of 1219 SSRs in 20 representative melon varieties (lines), with 470 polymorphic SSRs selected. Eighteen SSR markers with rich contents of polymorphism information (PIC) were selected as the core SSRs to establish the DNA fingerprint database. The utility of this core set of SSRs was further demonstrated and validated in 471 melon accessions including commercial cultivars and elite lines.

## Materials and Methods

### Plant Materials

Four hundred and seventy-one melon varieties (lines) were employed in this study (Table S1). Plant materials were obtained mainly from the major melon producing areas of China, covering 20 provinces, municipalities, and autonomous regions. A small number of melon varieties (lines) originated from Japan, the United States, India, and some other countries which further broadened the representativeness of target populations. These 471 melon materials comprised two categories: thin rind melon (294) and thick rind melon (177). According to botanical classification, subspecies included *C. melo* subsp. *agrestis* (Naud) Greb (2 materials), *C. melo* subsp. *dudaim* (L.) Greb (one), *C. melo* subsp. *flexuosus* (L.) Greb (7), *C. melo* subsp. *conomon* (Thunb.) Greb (284) and *C. melo* subsp. *melo* Pang (177).

Three hundred and forty-five commercial cultivars were obtained directly from seed market vendors. A small number of commercial cultivars were from researchers who commissioned us to determine if plants with the same identifier were from different genetic lines. Of the remaining 126 breeding elite lines, outside breeders and researchers provided some, and others were our own stock kept in the laboratory. All the tested melon varieties were grown in the greenhouse at 25/18°C day/night.

### Strategies for Developing a Core Set of SSR Markers in Melon

Twenty melon genotypes with diverse genetic backgrounds and horticultural traits were screened to develop a core set of SSR markers (Table 1). These were chosen as representatives of the melon collection for maximum diversity. The starting marker set contained 1219 SSRs, for developing the core set of SSRs. Our objective was to eliminate sequentially the SSR markers with low power of discrimination to achieve a final combination of markers that could be used for DNA fingerprinting and genetic diversity analysis. The 1219 SSRs were obtained from the literature [26]–[32], online databases (NCBI Expressed Sequence Tags database, http://www.ncbi.nlm.nih.gov/dbEST/; the Curcurbit Genomics Database, http:/www.icugi.org/) and the Chinese Academy of Agricultural Sciences.

The 20 (maximum diversity) melon genotypes were screened for the 1219 SSRs. A final core set of SSRs was determined based on the following criteria: 1) a PIC value ≥0.55, 2) SSRs were evenly distributed across the melon genome with ≥one marker from each linkage group, 3) easy to use in a PCR assay (i.e., a clear PCR product under standard reaction conditions), and 4) each SSR detected one and only one locus.

**Table 1:** Characteristics of 20 melon varieties used for developing the melon DNA fingerprinting core SSRs.

No.	Name	Systematics	Fruit shape	Rind features	Flesh color	Fruit weight (g)	Soluble solid content (Brix)
1	Little Melon (wild)	*C. melo* ssp. *agrestis*	Round	Green	White	15.63	5
2	Play Melon	*C. melo* ssp. *dudaim*	Round	Yellow	Yellow	43.11	12.5
3	Qingpi Caigua No.1	*C. melo* ssp. *flexuosus*	Elongated	Green	White	360.09	5.4
4	Huapi Sushaogua	*C. melo* ssp. *conomon*	Elongated	Green, dark green stripes	Green	176.62	8.2
5	Heipi Sugua	*C. melo* ssp. *conomon*	Elongated	Dark green	Green	730.49	3.3
6	No.20	*C. melo* ssp. *conomon*	Oblate	Green	White	153.7	6.2
7	3-2-2	*C. melo* ssp. *conomon*	Oblong	Green, white stripes	White	261.24	12.3
8	7-1-1-2	*C. melo* ssp. *conomon*	Oblong	Green, dark green stripes	Green	215.32	7.7
9	13-4-6-1	*C. melo* ssp. *conomon*	Oblong	Yellow, white stripes	White	334.82	8.6
10	16-8-1-1	*C. melo* ssp. *conomon*	Oblong	Yellow, white stripes	Orange	307.45	9.5
11	Tianshuai (parent)	*C. melo* ssp. *conomon*	Oblong	White	White	198.83	9.2
12	Bailangua	*C. melo* ssp. *melo*	Round	White	Green	536.54	11.5
13	Tiedanzi	*C. melo* ssp. *melo*	Round	Orange	Green	388.75	11.3
14	Hongxincui	*C. melo* ssp. *melo*	Oblong	Grey-white	Red orange	803.21	12.8
15	Kalakesai	*C. melo* ssp. *melo*	Round	Green, dark green stripes	Red orange	613.05	12.1
16	PMR45	*C. melo* ssp. *melo*	Round	Densely netted	Red orange	353.92	12.3
17	TopMark	*C. melo* ssp. *melo*	Round	Densely netted	Orange	388.04	6.2
18	WI998	*C. melo* ssp. *melo*	Oblate	Orange, netted	Red orange	374.26	7.3
19	Elizabeth Male Parent	*C. melo* ssp. *melo*	Round	Yellow	Green	188.88	12.3
20	Yourangsika	*C. melo* ssp. *melo*	Round	Orange, crazing	White	222.76	10.6

### SSR Marker Analysis

For each melon variety, young tender leaves were collected from five 13-day-old seedlings, and stored at –80°C for DNA isolation as recommended by Luan et al. [33]. PCR reactions were performed in accordance with the procedures described by Luan et al. [34], with modifications. Briefly, each 15-µL PCR reaction mixture contained 15 ng template DNA, 0.4 µM each of the left and right primers, 2 mM MgCl_2_, 0.2 mM each of dNTPs, and 1 U *Taq* DNA polymerase in 1× PCR buffer (Takara, China). The PCR reaction began with 94°C for 5 min; then 35 cycles of 94°C for 30 s, 55°C for 30 s, and 72°C for 30 s; and a final extension at 72°C for 7 min. The PCR products were analyzed via 6% polyacrylamide gel electrophoresis in 1× TBE buffer. The gel was silver-stained using the Silver Sequence DNA Sequencing System (Promega, Madison, WI, USA).

### Data Analysis

Polymorphic SSR markers were scored for the presence or absence of corresponding bands among the tested accessions. Stutter and background bands were excluded. The scores ‘1’ and ‘0’ indicated the presence and absence of the bands, respectively. The PIC of each marker was calculated in accordance with Kirst et al. [35] as:
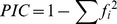
where *f_i_* is the band frequency of the *i* ^th^ allele.

Genetic similarities between melon varieties were calculated using Nei and Li’s coefficients index [36] with Free-Tree software [37]. A dendrogram was constructed with MEGA5 [38] software using the unweighted pair-group method with arithmetic mean (UPGMA).

### Representation of DNA Fingerprinting

The coding of fingerprints was expressed in accordance with Wang et al. [39] and Ma et al. [40]. Core SSRs were sorted in a fixed order, and expressed in capital letters. Each pair of SSR primers amplified polymorphic bands, and the amplicon sizes of variable alleles were recorded (in base pairs [bp]), and if the amplicon of a core SSR was polymorphic in one material, the polymorphic amplicon sizes were sorted in descending order and connected by a dash (i.e., “-”). For example, the fingerprinting code A100B300-200C0 means the variety has an amplicon of 100 bp at SSR locus A, amplicons of 300 bp and 200 bp at SSR locus B, and no amplicon at SSR locus C.

## Results

### Development of the Core Set of SSR Markers for DNA Fingerprinting

In total, 1219 SSRs were screened from 20 melon genotypes of diverse genetic backgrounds and horticultural traits, and 470 polymorphic SSRs were screened out. Taking into account PIC values, genomic distribution uniformity, PCR amplification efficiency, and other factors relevant to SSRs, finally 18 polymorphic SSRs were selected as the core set of SSRs (Table 2). Each pair of these selected SSR primers could detect varying numbers of polymorphic bands (range, 4 to 14; mean, 9). The PIC value ranged between 0.55 and 0.82, with an average of 0.68. Their amplified bands were legible, easy to count, and distinguishable from one another. These markers were, for the most part, evenly distributed across the melon genome.

**Figure 1:**
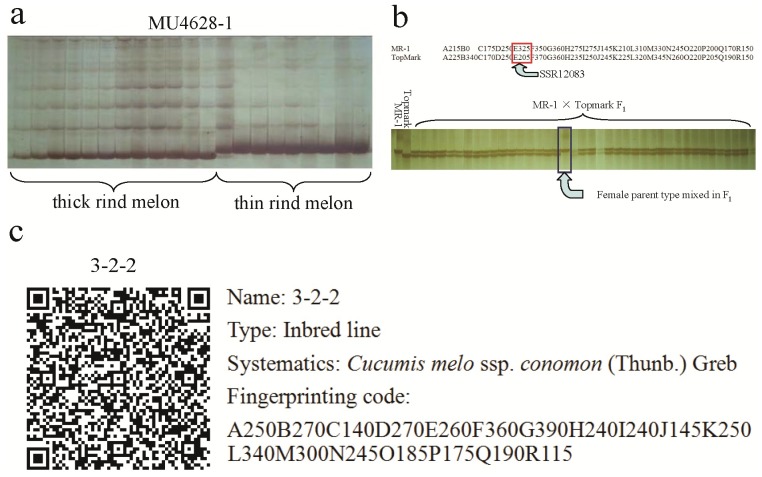
Application of the core set of SSR primers and the expression of melon DNA fingerprinting QR Code. **a.** SSR primer MU4628-1 could distinguish the thin rind melon and the thick rind melon. In this study, the total number of SSR primer pairs which could distinguish thin rind melon and thick rind melon clearly was 52. **b.** Each pair of the core SSR primers could be used in seed purity tests. In this example, fingerprinting codes of MR-1 and Topmark were analyzed. We found that SSR E (SSR12083, labeled by a red frame) had the bigger amplicon length difference in the two parents, so we chose SSR12083 to identify F_1_ seed purity. Most of the F_1_ were parent hybrid types, while few of them were female parent type (labeled by a blue frame). **c.** The DNA fingerprinting QR code of melon line 3-2-2 is at the left. This picture could be scanned by computers or a mobile device, such as a smartphone, PDA and so on. The right side shows the translation result of the left picture after scanning.

Of the 1219 SSRs, there were 52 SSRs which could differentiate thin rind from thick rind melon, as exemplified by the SSR primer MU4628-1 (Figure 1a). Although some of them matched well according to the screening criteria of the core set of SSRs, they were not selected based on visible phenotypic traits.

**Table 2:** The 18 core SSRs for DNA fingerprinting.

No.	Name	Primer sequences (5'-3', forward/reserve)	PIC	Size (bp)	Bands	Linkage group				Code name of SSRs
						A 1	B 2	C 3	D 4	
1	SSR12833	TCCCGACCTCTTCACGTAAC/GGAAGGCTCATACAGTGGGA	0.82	180–350	12	V	I	–	–	A
2	CMBR088	CCACTAAAGTTTCCTTATGTTTTGG/TGGTTGAGGAAGACTACCATCC	0.82	255–460	14	–	–	VII	X	B
3	CMBR052	CAGCGATGATCAACAGAAACA/GGCTGACACTCCCTGTACCT	0.82	100–215	14	–	–	VII	VII	C
4	GCM548	AACAGGTAGAGGAAAGCATG/TGACCCACTAGTACATCTCTC	0.78	200–330	12	–	XVI	II	–	D
5	SSR12083	GAATTGGCCCATCCTTCATT/GCCATTCCAAAAACTTTTCAAC	0.74	195–400	10	–	VI	–	XI	E
6	CMBR097	CGACAATCACGGGAGAGTTT/CATATTAGACCCATATTTGTTGCAT	0.72	340–400	7	–	XII	XII	XII	F
7	MU4104-1	TTTCCCGCATTGATTTTCTC/GAGAAACGCTTCCCACAAAC	0.71	275–440	8	IV	VII	–	I	G
8	NR38	TAAAACACTCTCGTGACTCC/GATCTGAGGTTGAAGCAAAG	0.69	225–360	8	XIX	XI	–	–	H
9	ECM147	GAAAGGTAGGAAGAAAGTGAAGA/ACTCTTGAAGCTGACCGATG	0.67	230–275	5	–	–	XI	XI	I
10	TJ138	AAAATGAAAACTCTTCGGCAAG/AAAACCCTTCTTGCCCTTGT	0.67	140–250	8	XVII	IV	–	I	J
11	CMBR002	TGCAAATATTGTGAAGGCGA/AATCCCCACTTGTTGGTTTG	0.65	165–295	9	–	–	VI	–	K
12	CM26	CCCTCGAGAAACCAGCAGTA/CACCTCCGTTTTTCATCACC	0.63	300–360	8	–	II	–	VII	L
13	MU9175-1	CAATTTCCAATCCATCTGCTC/ATCGAAATTCCTCCCTCGTT	0.63	295–345	8	–	VIII	–	II	M
14	MU5554-1	CCTTCATGATCCTCTACTAAACCC/TCTTCCATGCTTTTCTCGCT	0.60	230–290	8	–	XI	–	VI	N
15	SSR00398	ATTCAAACCCCGTTTAACCC/AGTGAAAATGGCGGAAACTG	0.60	185–340	11	–	XIII	–	–	O
16	ECM150	ACACACCTAATCTCCCTACCTTC/CTCAAACAACGTCAGCTGGT	0.59	170–205	8	–	–	IX	IX	P
17	TJ10	ACGAGGAAAACGCAAAATCA/TGAACGTGGACGACATTTTT	0.57	170–195	4	IX	V	III	–	Q
18	CMBR154	GATTCTTCCTCCTTCTAAAGGATA/AATGTGGGTGAGAGGACATT	0.55	100–180	8	–	XVIII	IV	–	R

1 Zhu et al. [41].

2 Gao et al. [42].

3 Diaz et al. [43] (ICUGI, http://www.icugi.org/).

4 Localization on melon genome [44] using SSR primer sequences by BLAST.

–, not on the linkage group.

Each pair of the core set of SSR primers could be used to identify the purity of F_1_ seeds (Figure 1b). For example (described in Figure 1b), we analyzed the fingerprinting codes of MR-1 and Topmark. SSR E (SSR12083, labeled with a red frame) had the bigger amplicon length difference in two parents, so SSR12083 was chosen to identify the purity of F_1_. Most of the F_1_ were parent hybrid types, and few of them were the female parent type (labeled with a blue frame). This feature could be used in purity tests of hybrid seeds.

### DNA Fingerprinting and Genetic Diversity Analysis of 471 Melon Materials

All of the 471 melon materials were subjected to polymorphism and genotyping screening using the core set of melon DNA fingerprinting SSR primers. In the present study, we used the SSR label plus the PCR amplicon length to represent the fingerprint code of each germplasm, and every fingerprint code corresponded to a long string.

The original purpose for melon germplasm DNA fingerprinting was to quickly gain genetic information for each melon line, input it into a computer database, and then be able to realize the fast alignment of genetic information of varieties of melon materials. Text information is difficult to read quickly from a computer, so the string fingerprinting code was transposed into graphical coding, the Quick Response (QR) barcode, which can be quickly scanned into the computer. Figure 1c is an example of the melon DNA fingerprinting QR code, which contains DNA fingerprinting, variety name, variety type and other information. The DNA fingerprinting QR code can be quickly scanned by computer or mobile operating systems. The QR code could thus be printed onto the packages of different varieties to quickly identify the contents for buyers and accreditation bodies, or be pasted onto seed bags or labels in experimental fields to enable the rapid identification of the germplasm, to support breeding material management and germplasm secrecy for breeders.

The fingerprint codes of these melon materials are listed in Table S2. The number of the amplified bands in these 471 melon materials ranged between 17 and 31, with an average of 21.46.

**Figure 2:**
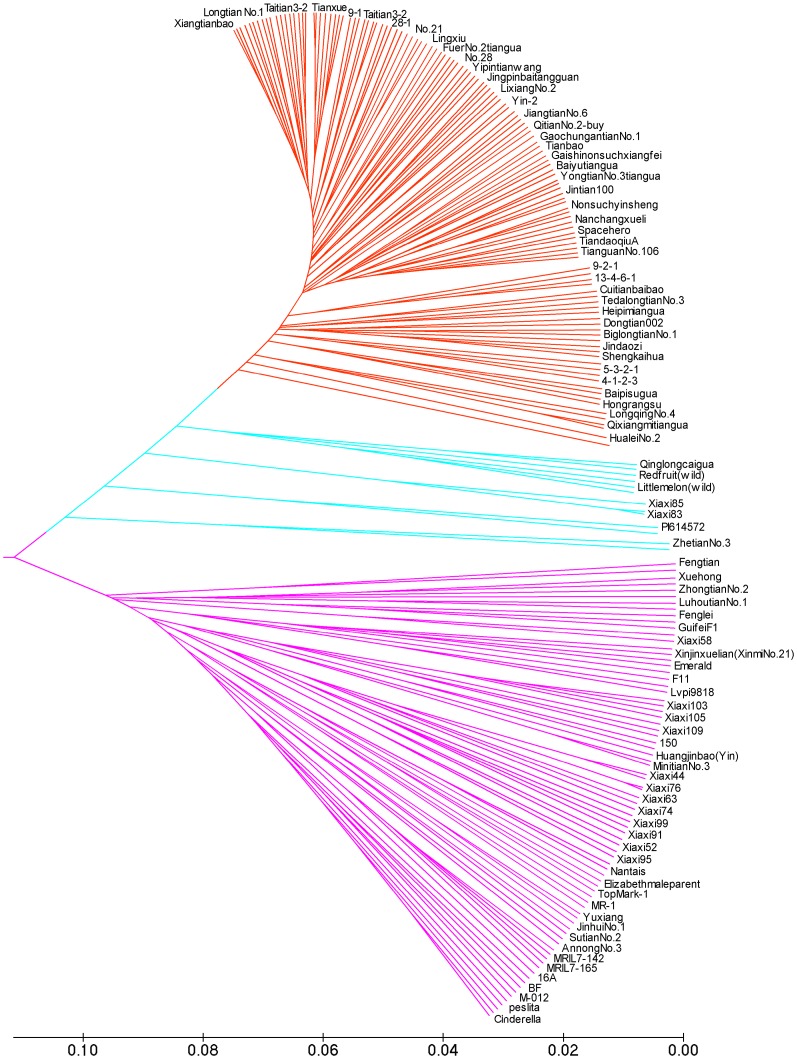
UPGMA dendrogram of 471 melon accessions based on the 18 core set of SSR markers. Materials with the same fingerprinting codes had been merged. The end of each branch represents a melon variety, and many names of varieties had been omitted due to space considerations. For more detailed classification information, see Figure S1. The 471 test accessions were in 3 distinct clusters: color codes red, blue, and pink.

The dendrogram of the 471 melon entries included in this study was based on UPGMA analysis, using a similarity matrix generated by the Nei and Li coefficient after amplification with 18 pairs of microsatellite primers (Figure 2). The resulting dendrogram had 3 distinct clusters. The first cluster (shown in red) consists only of *C. melo* subsp. *conomon* (Thunb.) Greb (284 varieties/lines). The second (blue) comprises *C. melo* subsp. *flexuosus* (L.) Greb (7 varieties/lines), *C. melo* subsp. *dudaim* (L.) Greb (1 variety), *C. melo* subsp. *agrestis* (Naud) Greb (2 varieties), and *C. melo* subsp. *melo* Pang (3 varieties). The third cluster (pink) contains only *C. melo* subsp. *melo* Pang (174 varieties/lines).

Interestingly, based on the screening by the core set of melon DNA fingerprinting SSR primers, there were 51 materials used in this study that could be separated into 17 groups and each group has the same fingerprinting codes, with the names and fingerprinting codes of these materials listed in Table 3. Further field traits survey results (data not shown) indicated that the materials with the same fingerprinting codes (each of these 17 groups) have also same or similar field characters. These materials could be considered as synonym materials.

**Figure 3:**
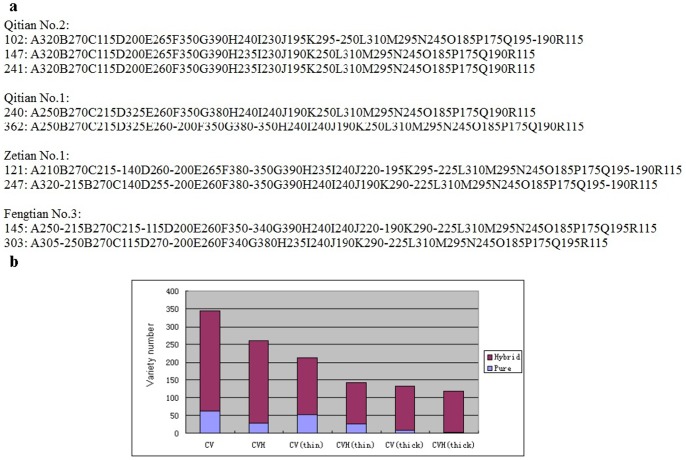
Alignment of homonym melon varieties and genetic purity detection of commercial melon varieties. a. Alignment of 4 groups of homonym melon varieties. Qitian No.1, Qitian No.2, Zetian No.1 and Fengtian No.3 represent four commercial varieties in which homonym phenomena were found. Numbers to the left of colons are the numbers of varieties in Table S2; fingerprinting codes are listed to the right of the colons. **b.** Genetic purity detection results of commercial melon varieties. CV: commercial cultivar; CVH: commercial cultivar labeled “hybrid” or “F_1_”; CV (thin): commercial thin rind melon cultivar; CVH (thin): commercial thin rind melon cultivar labeled “hybrid” or “F_1_”; CV (thick): commercial thick rind melon cultivar; CVH (thick): commercial thick rind melon cultivar labeled “hybrid” or “F_1_”.

Screening with the core set of SSR primers also identified four group of homonym commercial varieties: Qitian No.1, Qitian No.2, Zetian No.1, and Fengtian No.3 (Figure 3a). These are all well-known thin rind melon commercial varieties in China. On the other hand, some homozygous melon lines were detected in commercial melon varieties (Figure 3b), where the total number of commercial melon varieties was 345, and the number of identified pure materials was 62, or 18% of the total number of varieties. Even in the commercial cultivars labeled “hybrid” or “F_1_”, there were still 11% that were actually pure materials. This confusion was more common in thin rind melons than in thick rind–about 25% of thin rind melon varieties were identified as pure materials, while only about 7% of thick rind melon varieties were identified as pure. In the thick rind melon cultivars labeled “hybrid” or “F1”, all were hybrid cultivars. This could indicate that the thick rind melon seed market is more mature than the thin rind melon seed market.

**Table 3:** Materials with the same fingerprinting codes.

Group No.	Material Name	Fingerprinting code
1	No.22, 12-2-2-1, 14-1-3-1	A215B270C115D200E265F340G390H240I240J220K225L310M295N245O185P175Q195R115
2	28-1, 26-Yellow, 3-1-3, Wang-1-2, 03-1-3, Jingxuan Tiebaqing	A250B270C215D260E260F350G390H240I240J190K250L310M295N245O185P175Q195R115
3	Xiangtiancui, Zhentianmei, Lvzhou Tianbao, Gagatian	A250B270C215-115D260E260F350G390H240I240J220-195K250L310M295N245O185P175Q195-190R115
4	9-1, Taitian1-5-5, Taitian2-1-1, Taitian2-2-1, Taitian2-2-2, Taitian2-3-1, Taitian2-3-2, Taitian2-5-1	A250B270C140D305E265F350G390H235I240J190K250L310M295N245O185P175Q190R115
5	Qi-2-3-4, Taitian2-5-5	A250B270C215D330E265F350G380H240I240J190K250L310M295N245O185P175Q190R115
6	Taitian1-3-5, Taitian1-4-3	A215B270C140D200E260F340G390H240I240J190K250L310M295N245O185P175Q190R115
7	Zhen Xiangtian, Gaotang Prince, Mengtianbaibao, Super Xiangmiguawang, Jiangtian, Jingxuan Disease-resistant Hefeng No.5	A250B270C215D270E260F350-340G390H240I240J190K250L310M295N245O260-185P175Q195-190R115
8	Xiangtian No.1, Super Tianmi	A250B270C215D325E260F350G390H240-235I240J190K250L310M295N245-235O185P175Q195R115
9	Longbai No.1, Longtianwang	A320B270C115D270E265F390G390H240I240J195K250L310M295N245O185P175Q195R115
10	Tiangua, Koukoutian	A320-250B270C215-115D260E260F350G390H240I240J190K250L310M295N245O185P175Q195R115
11	Xiangpiaopiao, Jinshuai	A250-210B270C215-140D260E260F380-350G390H240I240J190K250L310M295N245O185P175Q195R115
12	Jingmei 009, Gaochun Rainbow No.7 F_1_	A320-250B270C140D260-200E260F350G390H240I240J190K250L310M295N245O185P175Q195R115
13	Jilinnongda No.8, Northeast Guawang	A320-215B270C145D200E250F350-340G390H240I240J190K290-225L310M295N245O185P175Q195R115
14	Xinfu No.19, Yate	A250B270C215-140D260E260F350G390H240I240J190K250L340-310M315-295N245O185P175Q195-190R115
15	Jinmi, Jinrui	A210-180B340C185-140D270-255E205F370-360G440-430H265I250J245-220K295-225L310M345-325N260O260P180Q170R180-115
16	Jingpin Xuemeiren, Balengcui	A320B270C140D200E265F350G395H235I240J195K250L310M295N245O185P175Q195R115
17	Big Longtian No.1, Big Hongchengcui	A320B270C115D260E265F350G395H235I240J195K250L310M295N245O185P175Q195R115

## Discussion

### Selection and Application of the Core Set of SSR Markers in Melon

In this study, 18 SSRs were selected from an initial 1219 as the core set of SSRs. The selected SSR markers have high PIC values, and are evenly distributed across the melon genome with at least one marker from each linkage group. They are easy to use in PCR assays under standard reaction conditions, and each SSR detects a single locus. The utility of this core set of SSRs was validated when used to analyze 471 melon accessions, including commercial cultivars and elite lines, and could sufficiently distinguish between cultivars and lines. Furthermore, results of the field trait survey indicated that materials with identical fingerprint codes (Table 3) based on these SSRs also shared the same or similar field characters, further validating the reliability of these SSRs. Importantly, each pair of the core set of SSR primers could establish the purity of F_1_ seeds (Figure 1b), and thus could be used for purity tests of hybrid seeds. This study is the first attempt to develop a core set of SSR markers to establish a melon DNA fingerprint database, and we will continue to monitor its application and validation and to improve it whenever necessary.

### Chinese Melon Diversity Analysis

In this study, we constructed a UPGMA dendrogram, which indicated that Chinese marketable melons could be divided into three distinct hierarchical clusters (Figure 2). Such a classification accords well with Chinese traditional melon classification, which divides melons into two groups by rind: thin rind (red, in the figure) and thick rind (pink). Wild melon, vegetable melon, and some kinds of thin-thick rind hybrids are in an intermediate position (blue) between the two groups above.

The thin rind melons had an average amplification band number of 20.61, less than that of the thick rind melons with an average number of 22.88. Furthermore, genetic similarities among the thin rind melons were 0.79–0.99, with an average of 0.94, which were higher than that of the thick rind melons (range 0.70–0.99, average 0.85). These results showed that the two groups are genetically distinct from each other, which might be because they have been developed independently for many years. Variations within genomic regions might be due to differences in selection pressure on target genes and traits between the two groups. The large difference between the average genetic diversity of the thin rind and thick rind melons could be because the thick rind melons in this study were collected from diverse regions, including southern, northern, northeastern, and Yangtze River regions of China, while most of the thin rind melons were collected from only northern and northeastern China. In addition, the genetic base of Chinese thin rind melon is relatively narrow. Therefore, detection and utilization of genetic variations and cultivar identification, and the expansion of genetic diversity are important tasks for Chinese thin rind melon breeders.

### Problems in Chinese Melon Breeding and the Seed Market

In this study, we found three main problems in the Chinese melon seed market. First, some varieties with the same parentage have different trade names. Second, there are varieties that have different parentage but carry the same name. Finally, some pure melon lines are sold as commercial varieties; there are even some commercial varieties that are labeled “hybrid” or “F_1_” but in fact are pure lines. Based on the screening by the core set of melon DNA fingerprinting SSR primers, 51 materials were reclassified into 17 groups and each group has the same fingerprinting codes, while these materials in each group also share the same or similar field characters. Thus these materials could be considered as synonym materials, and the majority of the synonym commercial melon cultivars are thin rind melon. On the other hand, we found 4 groups of homonym commercial varieties, which were all well-known thin rind melon commercial varieties in China: Qitian No.1, Qitian No.2, Zetian No.1 and Fengtian No.3 (See Figure 3a). These discrepancies, discovered through DNA fingerprinting, indicate that some seed producers or dealers have mislabeled produce in favor of the more popular sold, which infringes on the rights of melon breeders and seed production enterprises.

Another notable phenomenon was that not all varieties in Chinese melon seed markets were F_1_ generation hybrids. In a mature seed market, varieties sold should be hybrids. However, through the DNA fingerprinting, we found some pure melon lines were detected in commercial melon varieties(Figure 3b), and this phenomenon was more common in thin rind melon than in thick rind melon, indicating that the thick rind melon seed market could be more mature than thin rind melon seed market. The above results indicated that the melon market in China was still immature, especially for thin rind melon. One of the important reasons could be due to the narrow genetic resources of Chinese thin rind melon, which made the breeding relatively difficult. To solve this problem, efforts should be made to create new germplasm resources, while strengthening seed market management is also necessary.

## Supporting Information

Figure S1 Detailed UPGMA dendrogram of 471 melon accessions based on the 18 core set of SSR markers.
(PDF)

Table S1 Varieties (lines) employed in this study. Name: *materials used to screen SSRs; Type: L inbred or elite line, CV commercial cultivar, CV (H) commercial cultivar labeled with “hybrid” or “F_1_”; Systematics: A *Cucumis melo* ssp. *conomon* (Thunb.) Greb, B *Cucumis melo* ssp. *melo* Pang, C *Cucumis melo* ssp. *dudaim* (L.) Greb, D *Cucumis melo* ssp. *agrestis* (Naud) Greb, E *Cucumis melo* ssp. *flexuosus* (L.) Greb; Obtain way: K kept in our laboratory, B bought in the market, 1 Daqing Branches of Heilongjiang Academy of Agricultural Sciences, 2 Zhengzhou Fruit Research Institute of Chinese Academy of Agriculture Sciences, 3 Vegetable Research Institute of Shandong Academy of Agricultural Sciences, 4 Horticultural Research Institute of Guangxi Academy of Agricultural Sciences, 5 Ningbo Agricultural Scientific Research Institute, 6 Horticultural Research Institute of Anhui Academy of Agricultural Sciences, 7 Vegetable Research Institute of Gansu Academy of Agricultural Sciences, 8 Shanghai Jiading District Agro-Technology Extension Service Center, 9 Vegetable Research Institute of Jiangsu Academy of Agricultural Sciences, 10 Center of Hami Melon of Xinjiang Academy of Agricultural Sciences, 11 Horticultural Research Institute of Henan Academy of Agricultural Sciences, 12 The Institute of Vegetables and Flowers of Chinese Academy of Agricultural Sciences, 13 Institute of Germplasm Resources of Ningxia Academy of Agriculture and Forestry Sciences, O other ways.
(DOC)

Table S2 The fingerprint codes of varieties (lines) employed in this study.
(DOC)
