# Supplementary figures and images for: DNA Fingerprinting of Chinese Melon Provides Evidentiary Support of Seed Quality Appraisal
Source: PLoS One. 2012 Dec 20;7(12):e52431. doi: 10.1371/journal.pone.0052431 (PMC3527501; doi:10.1371/journal.pone.0052431)

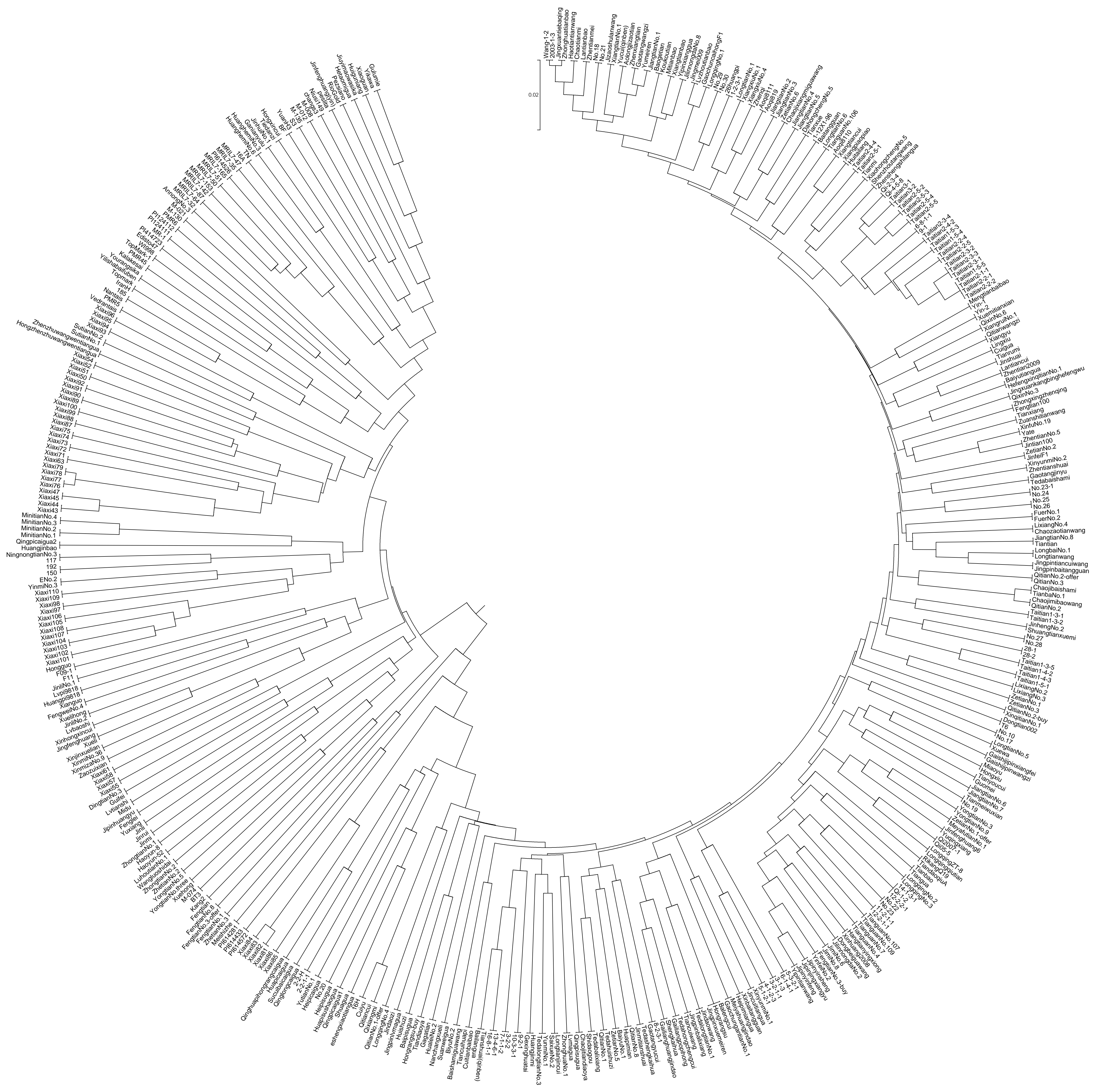

Supplement: Figure S1 — Detailed UPGMA dendrogram of 471 melon accessions based on the 18 core set of SSR markers. (PDF) [file pone.0052431.s001.pdf]
