# Supplementary material for: DNA Fingerprinting of Chinese Melon Provides Evidentiary Support of Seed Quality Appraisal
Source: PLoS One. 2012 Dec 20;7(12):e52431. doi: 10.1371/journal.pone.0052431 (PMC3527501; doi:10.1371/journal.pone.0052431)
Supplement: Table S1 — Varieties (lines) employed in this study. Name: *materials used to screen SSRs; Type: L inbred or elite line, CV commercial cultivar, CV (H) commercial cultivar labeled with “hybrid” or “F1”; Systematics: A Cucumis melo ssp. conomon (Thunb.) Greb, B Cucumis melo ssp. melo Pang, C Cucumis melo ssp. dudaim (L.) Greb, D Cucumis melo ssp. agrestis (Naud) Greb, E Cucumis melo ssp. flexuosus (L.) Greb; Obtain way: K kept in our laboratory, B bought in the market, 1 Daqing Branches of Heilongjiang Academy of Agricultural Sciences, 2 Zhengzhou Fruit Research Institute of Chinese Academy of Agriculture Sciences, 3 Vegetable Research Institute of Shandong Academy of Agricultural Sciences, 4 Horticultural Research Institute of Guangxi Academy of Agricultural Sciences, 5 Ningbo Agricultural Scientific Research Institute, 6 Horticultural Research Institute of Anhui Academy of Agricultural Sciences, 7 Vegetable Research Institute of Gansu Academy of Agricultural Sciences, 8 Shanghai Jiading District Agro-Technology Extension Service Center, 9 Vegetable Research Institute of Jiangsu Academy of Agricultural Sciences, 10 Center of Hami Melon of Xinjiang Academy of Agricultural Sciences, 11 Horticultural Research Institute of Henan Academy of Agricultural Sciences, 12 The Institute of Vegetables and Flowers of Chinese Academy of Agricultural Sciences, 13 Institute of Germplasm Resources of Ningxia Academy of Agriculture and Forestry Sciences, O other ways. (DOC) [file pone.0052431.s002.doc]

Table S1 Melon materials

| **No.** | **Name** | **Type** | **Systematics** | **Source** | **Obtain way** |
| --- | --- | --- | --- | --- | --- |
| 1 | No.10 | L | A | Heilongjiang, China | K |
| 2 | No.17 | L | A | Heilongjiang, China | K |
| 3 | No.18 | L | A | Heilongjiang, China | K |
| 4 | No.20* | L | A | Heilongjiang, China | K |
| 5 | No.21 | L | A | Heilongjiang, China | K |
| 6 | No.22 | L | A | Heilongjiang, China | K |
| 7 | No.23 | L | A | Heilongjiang, China | K |
| 8 | No.23-1 | L | A | Heilongjiang, China | K |
| 9 | No.24 | L | A | Heilongjiang, China | K |
| 10 | No.25 | L | A | Heilongjiang, China | K |
| 11 | No.26 | L | A | Heilongjiang, China | K |
| 12 | No.27 | L | A | Heilongjiang, China | K |
| 13 | No.28 | L | A | Heilongjiang, China | K |
| 14 | 28-1 | L | A | Heilongjiang, China | K |
| 15 | 28-2 | L | A | Heilongjiang, China | K |
| 16 | No.29 | L | A | Heilongjiang, China | K |
| 17 | No.30 | L | A | Heilongjiang, China | K |
| 18 | 26-Yellow | L | A | Heilongjiang, China | K |
| 19 | 1-2-3-1 | L | A | Heilongjiang, China | K |
| 20 | 2-2-1-1 | L | A | Heilongjiang, China | K |
| 21 | 2-2-H | L | A | Heilongjiang, China | K |
| 22 | 3-1-3 | L | A | Heilongjiang, China | K |
| 23 | 3-2-1-1 | L | A | Heilongjiang, China | K |
| 24 | 3-2-2* | L | A | Heilongjiang, China | K |
| 25 | 4-1-2-3 | L | A | Heilongjiang, China | K |
| 26 | 5-1-2-1 | L | A | Heilongjiang, China | K |
| 27 | 5-3-2-1 | L | A | Heilongjiang, China | K |
| 28 | 6-1-4-1 | L | A | Heilongjiang, China | K |
| 29 | 6-8-1-1 | L | A | Heilongjiang, China | K |
| 30 | 7-1-1-2* | L | A | Heilongjiang, China | K |
| 31 | 9-1 | L | A | Heilongjiang, China | K |
| 32 | 9-2-1 | L | A | Heilongjiang, China | K |
| 33 | 10-3-3-1 | L | A | Heilongjiang, China | K |
| 34 | 11-2-1-1 | L | A | Heilongjiang, China | K |
| 35 | 12-2-1-1 | L | A | Heilongjiang, China | K |
| 36 | 12-2-2-1 | L | A | Heilongjiang, China | K |
| 37 | 13-4-6-1* | L | A | Heilongjiang, China | K |
| 38 | 14-1-3-1 | L | A | Heilongjiang, China | K |
| 39 | 16-8-1-1* | L | A | Heilongjiang, China | K |
| 40 | Qi-1-2 | L | A | Heilongjiang, China | 1 |
| 41 | Qi-2-3-4 | L | A | Heilongjiang, China | 1 |
| 42 | Qi-4-5-8 | L | A | Heilongjiang, China | 1 |
| 43 | Wang-1-2 | L | A | Heilongjiang, China | K |
| 44 | 03-1-3 | L | A | Heilongjiang, China | K |
| 45 | Taitian1-3-1 | L | A | Heilongjiang, China | 1 |
| 46 | Taitian1-3-2 | L | A | Heilongjiang, China | 1 |
| 47 | Taitian1-3-5 | L | A | Heilongjiang, China | 1 |
| 48 | Taitian1-4-2 | L | A | Heilongjiang, China | 1 |
| 49 | Taitian1-4-3 | L | A | Heilongjiang, China | 1 |
| 50 | Taitian1-5-1 | L | A | Heilongjiang, China | 1 |
| 51 | Taitian1-5-3 | L | A | Heilongjiang, China | 1 |
| 52 | Taitian1-5-4 | L | A | Heilongjiang, China | 1 |
| 53 | Taitian1-5-5 | L | A | Heilongjiang, China | 1 |
| 54 | Taitian2-1-1 | L | A | Heilongjiang, China | 1 |
| 55 | Taitian2-2-1 | L | A | Heilongjiang, China | 1 |
| 56 | Taitian2-2-2 | L | A | Heilongjiang, China | 1 |
| 57 | Taitian2-2-4 | L | A | Heilongjiang, China | 1 |
| 58 | Taitian2-2-5 | L | A | Heilongjiang, China | 1 |
| 59 | Taitian2-3-1 | L | A | Heilongjiang, China | 1 |
| 60 | Taitian2-3-2 | L | A | Heilongjiang, China | 1 |
| 61 | Taitian2-3-3 | L | A | Heilongjiang, China | 1 |
| 62 | Taitian2-3-4 | L | A | Heilongjiang, China | 1 |
| 63 | Taitian2-4-2 | L | A | Heilongjiang, China | 1 |
| 64 | Taitian2-4-4 | L | A | Heilongjiang, China | 1 |
| 65 | Taitian2-5-1 | L | A | Heilongjiang, China | 1 |
| 66 | Taitian2-5-2 | L | A | Heilongjiang, China | 1 |
| 67 | Taitian2-5-3 | L | A | Heilongjiang, China | 1 |
| 68 | Taitian2-5-4 | L | A | Heilongjiang, China | 1 |
| 69 | Taitian2-5-5 | L | A | Heilongjiang, China | 1 |
| 70 | Taitian3-1 | L | A | Heilongjiang, China | 1 |
| 71 | Taitian3-2 | L | A | Heilongjiang, China | 1 |
| 72 | Yucui (parent) | L | A | Heilongjiang, China | K |
| 73 | Tianshuai (parent)* | L | A | Heilongjiang, China | K |
| 74 | Aolong Jizaotian | L | A | Heilongjiang, China | K |
| 75 | Suanweigua | L | A | Hainan, China | K |
| 76 | Nanchangxueli | L | A | Henan, China | K |
| 77 | Xinyunmi No.2 | CV | A | - | B |
| 78 | Zhen Tianshuai | CV | A | - | B |
| 79 | Zhen Xiangtian | CV | A | - | B |
| 80 | Gaotang Prince | CV | A | - | B |
| 81 | Jizaoshu Lanwang | CV | A | Jilin, China | B |
| 82 | Xiangtian No.1 | CV | A | - | B |
| 83 | Gaotang Jinyu | CV | A | - | B |
| 84 | Teda-Baishami | CV | A | - | B |
| 85 | Cuitian Baibao | CV | A | Tianjin, China | B |
| 86 | Tiancui Huapi | CV | A | Jilin, China | B |
| 87 | Baishami Guawang | CV | A | - | B |
| 88 | Baiyu No.2 | CV | A | - | B |
| 89 | Longbai No.1 | CV | A | - | B |
| 90 | Longtianwang | CV | A | - | B |
| 91 | Jingpin Tiancuiwang | CV | A | - | B |
| 92 | Jingpin Baitangguan | CV | A | - | B |
| 93 | Longtian Jincui | CV | A | - | B |
| 94 | Saixue No.2 | CV | A | Liaoning, China | B |
| 95 | Yunmi No.1 | CV | A | Liaoning, China | B |
| 96 | Teda Longtian No.3 | CV | A | Heilongjiang, China | B |
| 97 | Huangjinmi | CV | A | Shanxi, China | B |
| 98 | Gexinghuatai | CV | A | Liaoning, China | B |
| 99 | Super Baishami | CV | A | Heilongjiang, China | B |
| 100 | Tianba No.1 | CV | A | Liaoning, China | B |
| 101 | Super Mibaowang | CV | A | Heilongjiang, China | B |
| 102 | Qitian No.2 | CV | A | Heilongjiang, China | B |
| 103 | New Yunmi No.1 | CV | A | - | B |
| 104 | Jincuitiangua | CV | A | - | B |
| 105 | New Baitangguan | CV | A | - | B |
| 106 | Heipimiangua | CV | A | Henan, China | B |
| 107 | Super Tiandiaoya | CV | A | Heilongjiang, China | B |
| 108 | Qingpisugua | CV | A | Shandong, China | B |
| 109 | Mengtianbaibao | CV | A | Liaoning, China | B |
| 110 | Yin-1 | CV | A | - | B |
| 111 | Yin-2 | CV | A | - | B |
| 112 | Xuemitianxian | CV (H) | A | - | B |
| 113 | Jinheng No.2 | CV (H) | A | - | B |
| 114 | Shuangtianxuemi | CV (H) | A | - | B |
| 115 | Zhongxingzhenqing | CV (H) | A | - | B |
| 116 | Fengtian 100% | CV (H) | A | - | B |
| 117 | Lixiang No.4 | CV (H) | A | - | B |
| 118 | Chaozao Tianwang | CV (H) | A | - | B |
| 119 | Lixiang No.2 | CV (H) | A | - | B |
| 120 | Lixiang No.3 | CV (H) | A | - | B |
| 121 | Zetian No.1 | CV (H) | A | - | B |
| 122 | Zetian No.3 | CV (H) | A | - | B |
| 123 | Zetian No.6 | CV (H) | A | - | B |
| 124 | Super Xiangmiguawang | CV (H) | A | - | B |
| 125 | Yumeiren | CV (H) | A | - | B |
| 126 | Jiangtian No.1 | CV (H) | A | - | O |
| 127 | Jiangtian No.2 | CV (H) | A | - | O |
| 128 | Jiangtian No.3 | CV (H) | A | - | O |
| 129 | Jiangtian No.4 | CV (H) | A | - | O |
| 130 | Jiangtian No.5 | CV (H) | A | - | O |
| 131 | Jiangtian No.6 | CV (H) | A | - | O |
| 132 | Jiangtian No.7 | CV (H) | A | - | O |
| 133 | Jiangtian No.8 | CV (H) | A | - | O |
| 134 | Tiantian | CV (H) | A | - | O |
| 135 | Tianmi | CV (H) | A | - | O |
| 136 | Xiaohongcheng No.5 | CV (H) | A | - | O |
| 137 | Dahongcheng No.5 | CV (H) | A | - | O |
| 138 | Tianxue | CV (H) | A | - | O |
| 139 | M1-12×M1-96 | CV (H) | A | Heilongjiang, China | O |
| 140 | Baitangguan | CV (H) | A | Heilongjiang, China | B |
| 141 | Tianbao | CV (H) | A | Heilongjiang, China | B |
| 142 | Tiangua | CV (H) | A | Heilongjiang, China | B |
| 143 | Green Magua | CV (H) | A | Heilongjiang, China | B |
| 144 | Zhonghua No.1 | CV (H) | A | Heilongjiang, China | B |
| 145 | Fengtian No.3 | CV (H) | A | Heilongjiang, China | B |
| 146 | Yinfei No.2 | CV (H) | A | Heilongjiang, China | B |
| 147 | Qitian No.2 | CV (H) | A | Heilongjiang, China | B |
| 148 | New Qitian No.1 | CV (H) | A | Heilongjiang, China | B |
| 149 | Longtian No.1 | CV (H) | A | Heilongjiang, China | B |
| 150 | Xiangxiu No.1 | CV (H) | A | Heilongjiang, China | B |
| 151 | Xiangxiu No.4 | CV (H) | A | Heilongjiang, China | B |
| 152 | Zhenqi | CV (H) | A | Heilongjiang, China | B |
| 153 | Aoqi 8111 | CV (H) | A | Heilongjiang, China | B |
| 154 | Aoqi 819 | CV (H) | A | Heilongjiang, China | B |
| 155 | Aoqi 8110 | CV (H) | A | Heilongjiang, China | B |
| 156 | Xiangtiancui | CV (H) | A | Heilongjiang, China | B |
| 157 | Xiangpiaopiao | CV (H) | A | Heilongjiang, China | B |
| 158 | Huitailang | CV (H) | A | Heilongjiang, China | B |
| 159 | Tianrumi | CV (H) | A | Heilongjiang, China | B |
| 160 | Jinshuai | CV (H) | A | Heilongjiang, China | B |
| 161 | Lantiancui | CV (H) | A | Heilongjiang, China | B |
| 162 | Zhentian 2009 | CV (H) | A | Heilongjiang, China | B |
| 163 | Mitianbao | CV (H) | A | Heilongjiang, China | B |
| 164 | Xiangtianbao | CV (H) | A | Heilongjiang, China | B |
| 165 | Lantianbao | CV (H) | A | Heilongjiang, China | B |
| 166 | Zhentianmei | CV (H) | A | Heilongjiang, China | B |
| 167 | Jingxuan Tiebaqing | CV (H) | A | Heilongjiang, China | B |
| 168 | Zhonghua Tianbao | CV (H) | A | Heilongjiang, China | B |
| 169 | Shenzhou Tangwang | CV (H) | A | Heilongjiang, China | B |
| 170 | Zhenshengshi Langua | CV (H) | A | Heilongjiang, China | B |
| 171 | Baiyu Tiangua | CV (H) | A | Heilongjiang, China | B |
| 172 | Hefeng New Qitian No.1 | CV (H) | A | Heilongjiang, China | B |
| 173 | Jingxuan Disease-resistant Hefeng No.5 | CV (H) | A | Heilongjiang, China | B |
| 174 | Qixin No.3 | CV (H) | A | Heilongjiang, China | B |
| 175 | Qixin No.6 | CV (H) | A | Heilongjiang, China | B |
| 176 | Xiangrui No.1 | CV (H) | A | Heilongjiang, China | B |
| 177 | Qitian Wangzi | CV (H) | A | Heilongjiang, China | B |
| 178 | Xiangyu | CV (H) | A | Heilongjiang, China | B |
| 179 | Longtian No.5 | CV (H) | A | Heilongjiang, China | B |
| 180 | Xuewa | CV (H) | A | Heilongjiang, China | B |
| 181 | Longtian No.6 | CV (H) | A | Heilongjiang, China | B |
| 182 | Tianguan No.106 | CV (H) | A | Heilongjiang, China | B |
| 183 | Tianguan No.107 | CV (H) | A | Heilongjiang, China | B |
| 184 | Tianguan No.109 | CV (H) | A | Heilongjiang, China | B |
| 185 | Tianguan No.7 | CV (H) | A | Heilongjiang, China | B |
| 186 | Tianguan No.4 | CV (H) | A | Heilongjiang, China | B |
| 187 | Rikang Q19 | CV (H) | A | Heilongjiang, China | B |
| 188 | Tiandaoqiu A | CV (H) | A | Heilongjiang, China | B |
| 189 | Jingmei 009 | CV (H) | A | Heilongjiang, China | B |
| 190 | Lvzhou Tianbao | CV (H) | A | Heilongjiang, China | B |
| 191 | Haotian Tianwang | CV (H) | A | Jilin, China | B |
| 192 | Super Tianmi | CV (H) | A | Jilin, China | B |
| 193 | Baogetian | CV (H) | A | Jilin, China | B |
| 194 | Koukoutian | CV (H) | A | Jilin, China | B |
| 195 | Yipin Xianggua | CV (H) | A | Jilin, China | B |
| 196 | Jilinnongda No.8 | CV (H) | A | Jilin, China | B |
| 197 | Space Hero | CV (H) | A | Jilin, China | B |
| 198 | Xinhuang 2008 | CV (H) | A | Jilin, China | B |
| 199 | Northeast Guawang | CV (H) | A | Jilin, China | B |
| 200 | Jilinnongda No.2 | CV (H) | A | Jilin, China | B |
| 201 | Xinfu No.19 | CV (H) | A | Liaoning, China | B |
| 202 | Yate | CV (H) | A | Liaoning, China | B |
| 203 | Golden Phoenix No.6 | CV (H) | A | Liaoning, China | B |
| 204 | Yuqingxiang | CV (H) | A | Liaoning, China | B |
| 205 | Tiandiaoya | CV | A | Liaoning, China | B |
| 206 | Hongrangsu | CV | A | Liaoning, China | B |
| 207 | Huapi Sushaogua* | CV | A | Liaoning, China | B |
| 208 | Baipi Sugua | CV | A | Liaoning, China | B |
| 209 | Heipi Sugua* | CV | A | Liaoning, China | B |
| 210 | Huishuzi | CV | A | Liaoning, China | B |
| 211 | Sweet and crisp | CV (H) | A | Liaoning, China | B |
| 212 | Guomei | CV (H) | A | Liaoning, China | B |
| 213 | Gaishi Nonsuch Xiangfei | CV (H) | A | Liaoning, China | B |
| 214 | Gaishi Nonsuch Prince | CV (H) | A | Liaoning, China | B |
| 215 | Miaoyu | CV (H) | A | Liaoning, China | B |
| 216 | Hongxiu | CV (H) | A | Liaoning, China | B |
| 217 | Tianxiang | CV (H) | A | Liaoning, China | B |
| 218 | Diamond Tianwang | CV (H) | A | Beijing, China | B |
| 219 | Nonsuch Yinsheng | CV (H) | A | Beijing, China | B |
| 220 | Nonsuch Jingxiangyu | CV (H) | A | Beijing, China | B |
| 221 | Jingmi No.6 | CV (H) | A | Beijing, China | B |
| 222 | Jingmi No.8 | CV (H) | A | Beijing, China | B |
| 223 | Lingxiu | CV (H) | A | Beijing, China | B |
| 224 | Cuigua | CV (H) | A | Hebei, China | B |
| 225 | Hualei No.2 | CV (H) | A | Tianjin, China | B |
| 226 | Gagatian | CV (H) | A | Tianjin, China | B |
| 227 | Zhentian No.5 | CV (H) | A | Tianjin, China | B |
| 228 | Jintian 100 | CV (H) | A | Inner Mongolia, China | B |
| 229 | Nonsuch Yinfeng | CV (H) | A | Anhui, China | B |
| 230 | Yipin Tianwang | CV (H) | A | Anhui, China | B |
| 231 | Zetian No.2 | CV (H) | A | Heilongjiang, China | 1 |
| 232 | Jinfei F1 | CV (H) | A | Heilongjiang, China | 1 |
| 233 | Meiya Huangjindao | CV (H) | A | Heilongjiang, China | 1 |
| 234 | Gaochun Gantian No.1 | CV (H) | A | Heilongjiang, China | 1 |
| 235 | Gaochun Rainbow No.7 F_1_ | CV (H) | A | Heilongjiang, China | 1 |
| 236 | Longqing No.1 | CV (H) | A | Heilongjiang, China | 1 |
| 237 | Longqing No.2 | CV (H) | A | Heilongjiang, China | 1 |
| 238 | Longqing No.3 | CV (H) | A | Heilongjiang, China | 1 |
| 239 | Longqing No.4 | CV (H) | A | Heilongjiang, China | 1 |
| 240 | Qitian No.1 Tiangua | CV (H) | A | Heilongjiang, China | 1 |
| 241 | Qitian No.2 Tiangua | CV (H) | A | Heilongjiang, China | 1 |
| 242 | Qitian No.3 Tiangua | CV (H) | A | Heilongjiang, China | 1 |
| 243 | Fuer No.1 Tiangua | CV (H) | A | Heilongjiang, China | 1 |
| 244 | Fuer No.2 Tiangua | CV (H) | A | Heilongjiang, China | 1 |
| 245 | Yongtian No.3 Tiangua | CV (H) | A | Heilongjiang, China | 1 |
| 246 | Yongtian No.9 Tiangua | CV (H) | A | Heilongjiang, China | 1 |
| 247 | Zetian No.1 Tiangua | CV (H) | A | Heilongjiang, China | 1 |
| 248 | Meiya Futian No.1 Tiangua | CV (H) | A | Heilongjiang, China | 1 |
| 249 | Qi 2007-1 Tiangua | CV (H) | A | Heilongjiang, China | 1 |
| 250 | Qi 05-5 Tiangua | CV (H) | A | Heilongjiang, China | 1 |
| 251 | Longqing ZT-8 Tiangua | CV (H) | A | Heilongjiang, China | 1 |
| 252 | Longqing Qiutian Tiangua | CV (H) | A | Heilongjiang, China | 1 |
| 253 | Qi Xiangmi Tiangua | CV (H) | A | Heilongjiang, China | 1 |
| 254 | Qitian Cuitiangua | CV (H) | A | Heilongjiang, China | 1 |
| 255 | Cuiyu | CV (H) | A | Henan, China | 2 |
| 256 | 16H | L | B | - | O |
| 257 | WI998* | L | B | U.S.A | O |
| 258 | 16A | L | B | - | O |
| 259 | Elizabeth Male Parent* | L | B | - | O |
| 260 | TN | L | B | Hebei, China | O |
| 261 | BF | L | B | Hebei, China | O |
| 262 | Yuan H3 | L | B | Hebei, China | O |
| 263 | S3 | L | B | Hebei, China | O |
| 264 | M-135 | L | B | Anhui, China | O |
| 265 | M-012 | L | B | Anhui, China | O |
| 266 | M-008 | L | B | Anhui, China | O |
| 267 | Changes 3 | L | B | Hebei, China | O |
| 268 | Niusi149 | L | B | - | O |
| 269 | Tiedanzi* | L | B | Gansu, China | K |
| 270 | Hetao Migua | CV | B | Gansu, China | K |
| 271 | Bailangua* | CV | B | Henan, China | O |
| 272 | Hongxincui* | CV | B | Henan, China | O |
| 273 | Kalakesai* | CV | B | Henan, China | O |
| 274 | PMR45* | L | B | Beijing, China | K |
| 275 | Jiuyimaowakasi | CV | B | Russia | B |
| 276 | Cinderella | CV | B | Russia | B |
| 277 | Yourangsika* | CV | B | Russia | B |
| 278 | Xiaoguan | CV | B | Russia | B |
| 279 | Yikawa | CV | B | Russia | B |
| 280 | Gulumie | CV | B | Russia | B |
| 281 | Rio Gold | CV | B | Russia | B |
| 282 | Pezsiano | CV | B | Russia | B |
| 283 | Peslita | CV | B | Russia | B |
| 284 | Golden Pheonix (Yin) | CV (H) | B | Xinjiang, China | B |
| 285 | Midu | CV (H) | B | Heilongjiang, China | B |
| 286 | Green Angel | CV (H) | B | Heilongjiang, China | B |
| 287 | Guifei F1 | CV (H) | B | Hebei, China | B |
| 288 | Dingtian No.3 | CV (H) | B | Inner Mongolia, China | B |
| 289 | Fenglei | CV (H) | B | Inner Mongolia, China | B |
| 290 | Nonsuch Huangyu | CV (H) | B | Anhui, China | B |
| 291 | Luhoutian No.1 | CV (H) | B | Shandong, China | 3 |
| 292 | Lucky-52 | CV (H) | B | Guangxi, China | 4 |
| 293 | Lucky-8 | CV (H) | B | Guangxi, China | 4 |
| 294 | Zhongtian No.1 | CV (H) | B | Henan, China | 2 |
| 295 | Zhongtian No.2 | CV (H) | B | Henan, China | 2 |
| 296 | Network Era | CV (H) | B | Henan, China | 2 |
| 297 | Xuehong | CV (H) | B | Henan, China | 2 |
| 298 | Yongtian No.3 | CV (H) | B | Zhejiang, China | 5 |
| 299 | Yongtian No.5 | CV (H) | B | Zhejiang, China | 5 |
| 300 | Zhetian No.2 | CV (H) | B | Zhejiang, China | 5 |
| 301 | Zhetian No.3 | CV (H) | B | Zhejiang, China | 5 |
| 302 | Fengtian No.1 | CV (H) | B | Anhui, China | 6 |
| 303 | Fengtian No.3 | CV (H) | B | Anhui, China | 6 |
| 304 | Fengtian No.8 | CV (H) | B | Anhui, China | 6 |
| 305 | Jinmi | CV (H) | B | Anhui, China | 6 |
| 306 | Jinrui | CV (H) | B | Anhui, China | 6 |
| 307 | Jinli | CV (H) | B | Anhui, China | 6 |
| 308 | Yuxiang | CV (H) | B | Anhui, China | 6 |
| 309 | Huanghemi No.6 | CV (H) | B | Gansu, China | 7 |
| 310 | Huanghemi No.3 | CV (H) | B | Gansu, China | 7 |
| 311 | Gantian Yulu | CV (H) | B | Gansu, China | 7 |
| 312 | Jinhui No.1 | CV (H) | B | Shanghai, China | 8 |
| 313 | Red Pearl Net | CV (H) | B | Jiangsu, China | 9 |
| 314 | Pearl Net | CV (H) | B | Jiangsu, China | 9 |
| 315 | Sutian No.1 | CV (H) | B | Jiangsu, China | 9 |
| 316 | Sutian No.2 | CV (H) | B | Jiangsu, China | 9 |
| 317 | Fengwei No.4 | CV (H) | B | Xinjiang, China | 10 |
| 318 | Xianguo | CV (H) | B | Xinjiang, China | 10 |
| 319 | Huangpi 9818 | CV (H) | B | Xinjiang, China | 10 |
| 320 | Lvpi 9818 | CV (H) | B | Xinjiang, China | 10 |
| 321 | Jinli No.2 (Little Jinli) | CV (H) | B | Xinjiang, China | 10 |
| 322 | Xuelihong | CV (H) | B | Xinjiang, China | 10 |
| 323 | Zaoxianzui | CV (H) | B | Xinjiang, China | 10 |
| 324 | Xinmi Hybridization No.9 (Huangzuixian) | CV (H) | B | Xinjiang, China | 10 |
| 325 | Xinmi No.36 (Golden Dragon) | CV (H) | B | Xinjiang, China | 10 |
| 326 | Xinji Xuelian (Xinmi No.21) | CV (H) | B | Xinjiang, China | 10 |
| 327 | Xueli | CV (H) | B | Xinjiang, China | 10 |
| 328 | Golden Pheonix (Xinmi No.28) | CV (H) | B | Xinjiang, China | 10 |
| 329 | Xinhongxincui (Xinmi No.24) | CV (H) | B | Xinjiang, China | 10 |
| 330 | Emerald | CV (H) | B | Xinjiang, China | 10 |
| 331 | Jinli No.1 (Big Jinli) | CV (H) | B | Xinjiang, China | 10 |
| 332 | F11 | CV (H) | B | Henan, China | 11 |
| 333 | F09-1 | CV (H) | B | Henan, China | 11 |
| 334 | Play Melon* | L | C | Henan, China | K |
| 335 | Red Fruit (wild) | L | D | Hainan, China | K |
| 336 | Little Melon (wild)* | L | D | Shandong, China | K |
| 337 | Qingpi Caigua No.1* | CV | E | Jiangsu, China | B |
| 338 | Heipi Caigua | CV | E | Henan, China | B |
| 339 | Yutian No.1 | CV (H) | A | Beijing, China | B |
| 340 | Tianmeiwuxian | CV (H) | A | Ningxia, China | B |
| 341 | No.19 | L | A | Heilongjiang, China | K |
| 342 | 8-2-3-1 | L | A | Heilongjiang, China | K |
| 343 | Gaotang Yucui | CV | A | Jilin, China | B |
| 344 | Teda Shengkaihua | CV | A | - | B |
| 345 | Jinmi Tianshuai | CV | A | Tianjin, China | B |
| 346 | Jindaowang | CV | A | Heilongjiang, China | B |
| 347 | Big Longtian No.1 | CV | A | Heilongjiang, China | B |
| 348 | Jingpi Lvmagua | CV | A | - | B |
| 349 | Jindaozi | CV | A | - | B |
| 350 | Jingpin Xuemeiren | CV | A | Jilin, China | B |
| 351 | Balengcui | CV | A | Liaoning, China | B |
| 352 | Hongrangsu | CV | A | Tianjin, China | B |
| 353 | Qitian No.8 | CV | A | Heilongjiang, China | O |
| 354 | Huapimian | CV | A | Henan, China | B |
| 355 | Jingpin Balixiang | CV | A | - | B |
| 356 | Tiancuiang | CV | A | Heilongjiang, China | B |
| 357 | Big Hongchengcui | CV | A | Heilongjiang, China | B |
| 358 | Improved Pohongpi | CV | A | Heilongjiang, China | B |
| 359 | Shengkaihua | CV | A | Jilin, China | B |
| 360 | Improved Huangjindao | CV | A | Heilongjiang, China | B |
| 361 | Big Huishuzi | CV | A | Heilongjiang, China | B |
| 362 | Qitian No.1 | CV | A | Heilongjiang, China | B |
| 363 | Big Balixiang | CV | A | Heilongjiang, China | B |
| 364 | Shidaogou | CV | A | Beijing, China | B |
| 365 | Baiyu No.1 | CV | A | Beijing, China | B |
| 366 | Zetian No.5 | CV (H) | A | Heilongjiang, China | B |
| 367 | Dongtian 002 | CV (H) | A | Heilongjiang, China | O |
| 368 | TopMark* | L | B | U.S.A | K |
| 369 | T6 | L | B | Hebei, China | O |
| 370 | Fengtian | CV (H) | B | Hebei, China | O |
| 371 | Kang 2 | L | B | Hebei, China | O |
| 372 | BT3 | CV (H) | B | Hebei, China | O |
| 373 | M-074 | L | B | Anhui, China | O |
| 374 | M-130 | CV (H) | B | Anhui, China | O |
| 375 | M-021 | L | B | Anhui, China | O |
| 376 | Annong No.3 | CV (H) | B | Anhui, China | O |
| 377 | MRIL7-32 | L | B | U.S.A | O |
| 378 | MRIL7-35 | L | B | U.S.A | O |
| 379 | MRIL7-47 | L | B | U.S.A | O |
| 380 | MRIL7-50 | L | B | U.S.A | O |
| 381 | MRIL7-51 | L | B | U.S.A | O |
| 382 | MRIL7-64 | L | B | U.S.A | O |
| 383 | MRIL7-87 | L | B | U.S.A | O |
| 384 | MRIL7-142 | L | B | U.S.A | O |
| 385 | MRIL7-153 | L | B | U.S.A | O |
| 386 | MRIL7-165 | L | B | U.S.A | O |
| 387 | PI614526 | L | B | U.S.A | O |
| 388 | PI614572 | L | B | U.S.A | O |
| 389 | PI614433 | L | B | U.S.A | O |
| 390 | PI614281 | L | B | U.S.A | O |
| 391 | Meishizhe | CV | B | Russia | B |
| 392 | Xiaxi 43 | CV (H) | B | Japan | B |
| 393 | Xiaxi 44 | CV (H) | B | Japan | B |
| 394 | Xiaxi 45 | CV (H) | B | Japan | B |
| 395 | Xiaxi 47 | CV (H) | B | Japan | B |
| 396 | Xiaxi 50 | CV (H) | B | Japan | B |
| 397 | Xiaxi 51 | CV (H) | B | Japan | B |
| 398 | Xiaxi 52 | CV (H) | B | Japan | B |
| 399 | Xiaxi 54 | CV (H) | B | Japan | B |
| 400 | Xiaxi 55 | CV (H) | B | Japan | B |
| 401 | Xiaxi 57 | CV (H) | B | Japan | B |
| 402 | Xiaxi 58 | CV (H) | B | Japan | B |
| 403 | Xiaxi 61 | CV (H) | B | Japan | B |
| 404 | Xiaxi 63 | CV (H) | B | Japan | B |
| 405 | Xiaxi 71 | CV (H) | B | Japan | B |
| 406 | Xiaxi 72 | CV (H) | B | Japan | B |
| 407 | Xiaxi 73 | CV (H) | B | Japan | B |
| 408 | Xiaxi 74 | CV (H) | B | Japan | B |
| 409 | Xiaxi 75 | CV (H) | B | Japan | B |
| 410 | Xiaxi 76 | CV (H) | B | Japan | B |
| 411 | Xiaxi 77 | CV (H) | B | Japan | B |
| 412 | Xiaxi 78 | CV (H) | B | Japan | B |
| 413 | Xiaxi 79 | CV (H) | B | Japan | B |
| 414 | Xiaxi 81 | CV (H) | B | Japan | B |
| 415 | Xiaxi 82 | CV (H) | B | Japan | B |
| 416 | Xiaxi 83 | CV (H) | B | Japan | B |
| 417 | Xiaxi 84 | CV (H) | B | Japan | B |
| 418 | Xiaxi 85 | CV (H) | B | Japan | B |
| 419 | Xiaxi 86 | CV (H) | B | Japan | B |
| 420 | Xiaxi 87 | CV (H) | B | Japan | B |
| 421 | Xiaxi 88 | CV (H) | B | Japan | B |
| 422 | Xiaxi 89 | CV (H) | B | Japan | B |
| 423 | Xiaxi 90 | CV (H) | B | Japan | B |
| 424 | Xiaxi 91 | CV (H) | B | Japan | B |
| 425 | Xiaxi 92 | CV (H) | B | Japan | B |
| 426 | Xiaxi 93 | CV (H) | B | Japan | B |
| 427 | Xiaxi 94 | CV (H) | B | Japan | B |
| 428 | Xiaxi 95 | CV (H) | B | Japan | B |
| 429 | Xiaxi 96 | CV (H) | B | Japan | B |
| 430 | Xiaxi 97 | CV (H) | B | Japan | B |
| 431 | Xiaxi 98 | CV (H) | B | Japan | B |
| 432 | Xiaxi 99 | CV (H) | B | Japan | B |
| 433 | Xiaxi 100 | CV (H) | B | Japan | B |
| 434 | Xiaxi 101 | CV (H) | B | Japan | B |
| 435 | Xiaxi 102 | CV (H) | B | Japan | B |
| 436 | Xiaxi 103 | CV (H) | B | Japan | B |
| 437 | Xiaxi 104 | CV (H) | B | Japan | B |
| 438 | Xiaxi 105 | CV (H) | B | Japan | B |
| 439 | Xiaxi 106 | CV (H) | B | Japan | B |
| 440 | Xiaxi 107 | CV (H) | B | Japan | B |
| 441 | Xiaxi 108 | CV (H) | B | Japan | B |
| 442 | Xiaxi 109 | CV (H) | B | Japan | B |
| 443 | Xiaxi 110 | CV (H) | B | Japan | B |
| 444 | Yinmi No.3 (Yin) | CV (H) | B | Gansu, China | B |
| 445 | Russia No.2 (Yin) | CV (H) | B | Russia | O |
| 446 | Minitian No.1 | CV (H) | B | U.S.A | O |
| 447 | Minitian No.2 | CV (H) | B | U.S.A | O |
| 448 | Minitian No.3 | CV (H) | B | U.S.A | O |
| 449 | Minitian No.4 | CV (H) | B | U.S.A | O |
| 450 | Huangjinbao (Yin) | CV (H) | B | Heilongjiang, China | B |
| 451 | Qingpi Caigua 2 | CV | E | Anhui, China | B |
| 452 | Qinglong Caigua | CV | E | Hubei, China | B |
| 453 | Sucui Baicaigua | CV | E | Hebei, China | B |
| 454 | Huapi Caigua | CV | E | Anhui, China | B |
| 455 | Qinghuapi Hongrang Caigua | CV | E | Shandong, China | B |
| 456 | IranH | L | B | Iran | K |
| 457 | Topmark | L | B | U.S.A | K |
| 458 | Vedrantais | L | B | France | K |
| 459 | PMR5 | L | B | U.S.A | K |
| 460 | Edisto47 | L | B | U.S.A | K |
| 461 | PI414723 | L | B | India | K |
| 462 | MR-1 | L | B | India | K |
| 463 | PI124111 | L | B | India | K |
| 464 | PI124112 | L | B | India | K |
| 465 | PMR6 | L | B | India | K |
| 466 | Nantais | L | B | France | K |
| 467 | 185 | CV (H) | B | Beijing, China | 12 |
| 468 | 150 | CV (H) | B | Beijing, China | 12 |
| 469 | 192 | CV (H) | B | Beijing, China | 12 |
| 470 | 117 | CV (H) | B | Beijing, China | 12 |
| 471 | Ningnongtian No.3 | CV (H) | B | Ningxia, China | 13 |
