# Supplementary material for: DNA Fingerprinting of Chinese Melon Provides Evidentiary Support of Seed Quality Appraisal
Source: PLoS One. 2012 Dec 20;7(12):e52431. doi: 10.1371/journal.pone.0052431 (PMC3527501; doi:10.1371/journal.pone.0052431)
Supplement: Table S2 — The fingerprint codes of varieties (lines) employed in this study. (DOC) [file pone.0052431.s003.doc]

**Table S2** SSR DNA fingerprinting codes of 471 melon materials

| **No.** | **Name** | **SSR DNA fingerprinting codes** |
| --- | --- | --- |
| 1 | No.10 | A215B270C140D200E270F340G390H240I240J220K225L340M295N245O185P175Q195R115 |
| 2 | No.17 | A250B270C140D260E325F350G390H235I240J190K225L310M295N235O185P175Q195R115 |
| 3 | No.18 | A320B270C215D260E260F340G380H240I240J190K250L350M295N245O185P175Q195R115 |
| 4 | No.20* | A215B270C115D200E265F340G390H240I240J190K225L340M295N245O185P175Q195R115 |
| 5 | No.21 | A250B270C215D200E260F350G390H240I240J190K250L310M295N245O185P175Q195R115 |
| 6 | No.22 | A215B270C115D200E265F340G390H240I240J220K225L310M295N245O185P175Q195R115 |
| 7 | No.23 | A250B270C140D200E260F350G390H235I240J190K250L310M295N245O185P175Q195R115 |
| 8 | No.23-1 | A320B270C215D260E265F350G390H240I240J190K250L310M295N245O185P175Q195R115 |
| 9 | No.24 | A320B270C215D260E260F350G390H240I240J220K295L310M295N245O185P175Q190R0 |
| 10 | No.25 | A320B270C140D260E260F390G390H240I240J220K250L310M295N245O185P175Q190R115 |
| 11 | No.26 | A215B270C115D200E265F340G390H240I240J190K225L310M295N245O185P175Q195R115 |
| 12 | No.27 | A210B270C115D260E260F350G390H265I240J190K225L340M295N245O185P175Q190R115 |
| 13 | No.28 | A250B270C140D325E265F350G380H240I240J190K250L310M295N245O185P175Q190R115 |
| 14 | 28-1 | A250B270C215D260E260F350G390H240I240J190K250L310M295N245O185P175Q195R115 |
| 15 | 28-2 | A215B270C215D305E260F340G390H240I240J190K250L310M325N245O185P175Q195R115 |
| 16 | No.29 | A215B270C215D200E260F340G390H240I240J190K250L310M295N245O185P175Q195R115 |
| 17 | No.30 | A250B270C215D260E265F350G390H240I240J190K250L340M295N245O185P175Q195R115 |
| 18 | 26-Yellow | A250B270C215D260E260F350G390H240I240J190K250L310M295N245O185P175Q195R115 |
| 19 | 1-2-3-1 | A250B440C215D260E260F370G390H270I240J190K250L310M295N235O185P175Q195R115 |
| 20 | 2-2-1-1 | A320B300C115D330E260F390G390H235I230J190K250L340M295N245O185P175Q195R115 |
| 21 | 2-2-H | A215B300C115D330E260F390G390H235I230J190K250L340M325N245O185P175Q195R115 |
| 22 | 3-1-3 | A250B270C215D260E260F350G390H240I240J190K250L310M295N245O185P175Q195R115 |
| 23 | 3-2-1-1 | A215B270C115D200E260F340G390H240I240J220K225L310M295N245O185P175Q190R0 |
| 24 | 3-2-2* | A250B270C140D270E260F360G390H240I240J145K250L340M300N245O185P175Q190R115 |
| 25 | 4-1-2-3 | A215B270C115D200E250F340G390H240I240J220K225L310M295N245O185P175Q195R115 |
| 26 | 5-1-2-1 | A250B300C215D330E260F380G390H235I240J195K250L310M325N245O185P175Q190R115 |
| 27 | 5-3-2-1 | A215B270C115D200E270F340G390H240I240J220K225L0M295N245O185P175Q195R115 |
| 28 | 6-1-4-1 | A0B270C140D330E250F380G390H240I240J190K250L340M295N0O260P185Q190R0 |
| 29 | 6-8-1-1 | A250B270C215D270E260F350G390H240I240J190K250L310M295N245O185P175Q195R115 |
| 30 | 7-1-1-2* | A320B300C140D270E250F380G390H240I240J145K250L350M325N245O185P175Q195R115 |
| 31 | 9-1 | A250B270C140D305E265F350G390H235I240J190K250L310M295N245O185P175Q190R115 |
| 32 | 9-2-1 | A210B270C140D305E260F380G390H240I240J195K250L310M325N245O185P175Q190R0 |
| 33 | 10-3-3-1 | A215B270C115D200E265F340G390H240I240J190K290L340M295N245O185P175Q195R115 |
| 34 | 11-2-1-1 | A250B270C215D200E260F350G390H240I240J220-190K225L310M295N0O260P185Q190R0 |
| 35 | 12-2-1-1 | A215B270C115D200E265F340G390H240I240J220K225L310M295N245O0P175Q195R115 |
| 36 | 12-2-2-1 | A215B270C115D200E265F340G390H240I240J220K225L310M295N245O185P175Q195R115 |
| 37 | 13-4-6-1* | A210B270C140D325E260F380G390H240I240J145K250L310M325N245O185P175Q190R115 |
| 38 | 14-1-3-1 | A215B270C115D200E265F340G390H240I240J220K225L310M295N245O185P175Q195R115 |
| 39 | 16-8-1-1* | A210B270C140D325E260F380G390H240I240J145K250L310M325N245O185P175Q190R115 |
| 40 | Qi-1-2 | A250B270C215D325E260F380G390H240I240J195K250L310M295N245O185P175Q190R115 |
| 41 | Qi-2-3-4 | A250B270C215D330E265F350G380H240I240J190K250L310M295N245O185P175Q190R115 |
| 42 | Qi-4-5-8 | A250B270C215D330E250F350G380H240I240J190K250L310M295N245O185P175Q190R115 |
| 43 | Wang-1-2 | A250B270C215D260E260F350G390H240I240J190K250L310M295N245O185P175Q195R115 |
| 44 | 03-1-3 | A250B270C215D260E260F350G390H240I240J190K250L310M295N245O185P175Q195R115 |
| 45 | Taitian1-3-1 | A320B270C115D200E260F380G390H235I240J195K250L310M295N245O185P175Q195R115 |
| 46 | Taitian1-3-2 | A215B270C140D305E265F350G390H240I240J190K250L310M295N245O185P175Q195R115 |
| 47 | Taitian1-3-5 | A215B270C140D200E260F340G390H240I240J190K250L310M295N245O185P175Q190R115 |
| 48 | Taitian1-4-2 | A250B270C140D200E260F370G390H240I250J245K290L340M300N245O185P175Q190R115 |
| 49 | Taitian1-4-3 | A215B270C140D200E260F340G390H240I240J190K250L310M295N245O185P175Q190R115 |
| 50 | Taitian1-5-1 | A0B270C140D305E265F350G390H235I240J190K250L310M295N245O185P175Q190R115 |
| 51 | Taitian1-5-3 | A250B270C140D305E265F350G390H240I240J190K250L310M295N245O185P175Q190R115 |
| 52 | Taitian1-5-4 | A250B270C215D305E265F350G390H240I240J195K250L310M295N245O185P175Q190R115 |
| 53 | Taitian1-5-5 | A250B270C140D305E265F350G390H235I240J190K250L310M295N245O185P175Q190R115 |
| 54 | Taitian2-1-1 | A250B270C140D305E265F350G390H235I240J190K250L310M295N245O185P175Q190R115 |
| 55 | Taitian2-2-1 | A250B270C140D305E265F350G390H235I240J190K250L310M295N245O185P175Q190R115 |
| 56 | Taitian2-2-2 | A250B270C140D305E265F350G390H235I240J190K250L310M295N245O185P175Q190R115 |
| 57 | Taitian2-2-4 | A250B270C140D200E265F350G380H240I240J190K250L310M295N245O185P175Q190R115 |
| 58 | Taitian2-2-5 | A250B270C215D305E265F350G390H240I240J190K250L310M295N245O185P175Q190R115 |
| 59 | Taitian2-3-1 | A250B270C140D305E265F350G390H235I240J190K250L310M295N245O185P175Q190R115 |
| 60 | Taitian2-3-2 | A250B270C140D305E265F350G390H235I240J190K250L310M295N245O185P175Q190R115 |
| 61 | Taitian2-3-3 | A250B270C215D305E265F350G390H235I240J190K250L310M295N245O185P175Q190R0 |
| 62 | Taitian2-3-4 | A250B270C215D200E265F350G390H240I240J190K250L310M295N245O185P175Q190R115 |
| 63 | Taitian2-4-2 | A250B270C0D260E265F370G390H240I240J190K250L310M295N245O185P175Q190R115 |
| 64 | Taitian2-4-4 | A250B270C140D305E265F350G390H240I240J190K250L310M295N245O185P175Q195R115 |
| 65 | Taitian2-5-1 | A250B270C140D305E265F350G390H235I240J190K250L310M295N245O185P175Q190R115 |
| 66 | Taitian2-5-2 | A250B270C140D330E265F350G380H240I240J190K250L310M295N245O185P175Q190R115 |
| 67 | Taitian2-5-3 | A250B0C215D330E265F350G380H240I240J190K250L310M295N245O185P175Q190R115 |
| 68 | Taitian2-5-4 | A250B270C140D330E265F350G380H240I240J190K250L310M295N245O185P175Q195R115 |
| 69 | Taitian2-5-5 | A250B270C215D330E265F350G380H240I240J190K250L310M295N245O185P175Q190R115 |
| 70 | Taitian3-1 | A250B270C215D305E265F380G380H240I240J190K250L310M295N245O185P175Q190R115 |
| 71 | Taitian3-2 | A320B270C215D200E265F380G390H235I240J190K250L310M295N245O185P175Q195R115 |
| 72 | Yucui (parent) | A250B270C215D270E260F350G390H240I240J190K250L310M295N245O185P175Q195R0 |
| 73 | Tianshuai (parent)* | A320B270C140D200E265F350G390H235I240J145K250L310M295N245O185P175Q195R115 |
| 74 | Aolong Jizaotian | A250B270C215D270E260F350G390H240I240J190K250L310M295N245O0P175Q195R115 |
| 75 | Suanweigua | A215B300C215D250E325F340G390H240I230J245K250L310M345N245O185P175Q195R180 |
| 76 | Nanchangxueli | A320B270C115D200E265F390G390H235I240J195K225L310M295N245O185P175Q195R115 |
| 77 | Xinyunmi No.2 | A215B460-270C115D200E265F340G390H240I240J190K250L310M295N245O185P175Q195R115 |
| 78 | Zhen Tianshuai | A250B270C215D270E260F350G390-380H240I240J190K250L310M295N245O185P175Q190R115 |
| 79 | Zhen Xiangtian | A250B270C215D270E260F350-340G390H240I240J190K250L310M295N245O260-185P175Q195-190R115 |
| 80 | Gaotang Prince | A250B270C215D270E260F350-340G390H240I240J190K250L310M295N245O260-185P175Q195-190R115 |
| 81 | Jizaoshu Lanwang | A320B270C215-115D270E265F350G390H235I240J190K250L310M295N245O185P175Q195R115 |
| 82 | Xiangtian No.1 | A250B270C215D325E260F350G390H240-235I240J190K250L310M295N245-235O185P175Q195R115 |
| 83 | Gaotang Jinyu | A250B420-270C215D270E260F350G380H240I240J190K250L310M295N245O185P175Q195R115 |
| 84 | Teda-Baishami | A320B420-270C140D300E250F380G380H240I240J190K250L310M295N245O185P175Q190R115 |
| 85 | Cuitian Baibao | A320B270C215-140D300E250F350G390H240I240J190K250L340-310M325N245O185P175Q190R115 |
| 86 | Tiancui Huapi | A215B270C140D200E250F400G360H235I240J190K290L340M325-295N245O185P175Q195R115 |
| 87 | Baishami Guawang | A215B440-270C115D200E265F340G390H240I240J220K225L340M295N245O185P175Q195R115 |
| 88 | Baiyu No.2 | A210B300-270C115D270E260F380G380H240I240J0K0L310M325-295N245O185P175Q195R0 |
| 89 | Longbai No.1 | A320B270C115D270E265F390G390H240I240J195K250L310M295N245O185P175Q195R115 |
| 90 | Longtianwang | A320B270C115D270E265F390G390H240I240J195K250L310M295N245O185P175Q195R115 |
| 91 | Jingpin Tiancuiwang | A250B270C215D270E260F350G390H245I240J190K250L310M295N245O185P175Q195R115 |
| 92 | Jingpin Baitangguan | A320B270C140D200E265F350G390H240I240J195K250L310M295N245O185P175Q195R115 |
| 93 | Longtian Jincui | A250B270C140D300E265F350G390H245I240J195K250L340M325N245O185P175Q190R115 |
| 94 | Saixue No.2 | A215B270C115D270E260F380G390H235I240J190K190L310M295N245O185P175Q195R115 |
| 95 | Yunmi No.1 | A250-215B270C115D200E260F340G380H245I240J190K250L310M295N245O185P175Q195R115 |
| 96 | Teda Longtian No.3 | A210B270C140D200E260F380G390H245I240J195K250L310M325N245O185P175Q190R0 |
| 97 | Huangjinmi | A215B0C170D255E265F360G390H245I230J190K250L310M295N245O185P175Q195R115 |
| 98 | Gexinghuatai | A215B450-270C140D330E260F380G390H240I240J190K250L340M0N245O185P175Q195R115 |
| 99 | Super Baishami | A250B270C215D330E260F350G380H245I240J190K250L310M295N235O185P175Q190R115 |
| 100 | Tianba No.1 | A250B270C215D330E265F350G380H245I240J190K250L310M295N245O185P175Q190R115 |
| 101 | Super Mibaowang | A250B270C215D330E250F350G380H245I240J190K250L310M295N245O260P175Q190R115 |
| 102 | Qitian No.2 | A320B270C115D200E265F350G390H240I230J195K0L310M295N245O185P175Q195-190R115 |
| 103 | New Yunmi No.1 | A215B270C115D200E265F340G380H240I240J190K250L310M295N245O185P175Q195R115 |
| 104 | Jincuitiangua | A215B450-270C170D255E265F340G390H270-240I230J195K290L310M345-295N245O185P175Q195R115 |
| 105 | New Baitangguan | A250B450-270C215D270E260F350G390H240I240J190K250L310M295N245O185P175Q195R115 |
| 106 | Heipimiangua | A215B270C160D325E325-260F380G380H235I240J190K250L350M295N245O185P175Q195R115 |
| 107 | Super Tiandiaoya | A215B300C140D300E250F380G390H240I240J195K250L350M325N245O185P175Q195R115 |
| 108 | Qingpisugua | A320B270C145D200E265F350G390H240I240J220K250L310M300N245O185P175Q170R115 |
| 109 | Mengtianbaibao | A250B270C215D270E260F350-340G390H240I240J190K250L310M295N245O260-185P175Q195-190R115 |
| 110 | Yin-1 | A0B270C145D200E265F350G390H240I240J220K250L310M300N245O185P175Q170R115 |
| 111 | Yin-2 | A250B270C215D300E325-260F350G390H240I240J190K250L310M295N245O185P175Q195R115 |
| 112 | Xuemitianxian | A320B270C115D270E265F380G390H240I240J195K250L310M295N245O185P175Q195R115 |
| 113 | Jinheng No.2 | A250B270C140D300E260F350G390H240I240J195K250L340M325-295N245O185P175Q195-190R115 |
| 114 | Shuangtianxuemi | A215B370-270C115D200E265F340G390H240I240J220K225L340M295N245O185P175Q195R115 |
| 115 | Zhongxingzhenqing | A250B270C215D300E260F350G390H240I240J190K250L310M295N245O185P175Q195R115 |
| 116 | Fengtian 100% | A250B270C140D0E260F350G390H240I240J195K250L340M325N245O185P175Q190R115 |
| 117 | Lixiang No.4 | A320B270C140D270E265F380-340G390H235I240J220-195K250L310M295N245O185P175Q195R115 |
| 118 | Chaozao Tianwang | A250B270C185D270E260F350G390H240I240J190K250L310M295N0O185P175Q195R115 |
| 119 | Lixiang No.2 | A320B270C140-115D270-260E265F380G390H235I240J220-195K250-225L340-310M295N245O185P175Q195R115 |
| 120 | Lixiang No.3 | A250B270C215D325-260E265F350-340G390H240I240-230J190K250L340-310M295N245O185P175Q195R115 |
| 121 | Zetian No.1 | A210B270C215-140D260-200E265F380-350G390H235I240J220-195K295-225L310M295N245O185P175Q195-190R115 |
| 122 | Zetian No.3 | A250-215B270C215D270-260E260F380-350G390H235I240J220-190K250L310M295N0O185P175Q190R115 |
| 123 | Zetian No.6 | A320-250B270C215-140D270-200E260F350G380H240I240J190K250L310M295N245O185P175Q195-190R115 |
| 124 | Super Xiangmiguawang | A250B270C215D270E260F350-340G390H240I240J190K250L310M295N245O260-185P175Q195-190R115 |
| 125 | Yumeiren | A250-215B270C215D270-200E260F380-350G390H240I240J190K250L310M295N245O185P175Q195R115 |
| 126 | Jiangtian No.1 | A250B270C215-140D270E260F380-350G390H240I240J190K250L310M325-295N245O185P175Q190R0 |
| 127 | Jiangtian No.2 | A250-215B270C215D330E270F380G390H240I240J220-190K250L310M295N245O185P175Q195R115 |
| 128 | Jiangtian No.3 | A320B270C140D270-200E265F350G390H240I240J190K250L310M295N245O185P175Q195R115 |
| 129 | Jiangtian No.4 | A250B270C215D270E260F350-340G390H240I240J190K250L310M295N245O260-185P175Q195-190R115 |
| 130 | Jiangtian No.5 | A215B270C115D270-200E265F380-340G390H235I240J220-190K190L340-310M295N245O185P175Q195R115 |
| 131 | Jiangtian No.6 | A250-215B270C215-115D200E260F340G390H240I240J220-195K250L310M295N245O185P175Q195R115 |
| 132 | Jiangtian No.7 | A320-250B270C215-140D270-200E265F350G390H240I240J190K250L310M295N245O185P175Q195R115 |
| 133 | Jiangtian No.8 | A320-250B270C115D270E265-260F380G390H240I240J190K250L310M325N245O185P175Q195-190R115 |
| 134 | Tiantian | A0B270C215-140D200E260F350G390H235I240J190K250L310M295N245O185P175Q195R115 |
| 135 | Tianmi | A320B270C140D200E265F350G390H235I240J190K250L310M295N245O185P175Q195R115 |
| 136 | Xiaohongcheng No.5 | A250B270C215D270E250F350G390H240I240J190K250L310M295N245O185P175Q195-190R115 |
| 137 | Dahongcheng No.5 | A0B270C215D270-200E265F350G390H240I240J190K250L310M295N245O185P185-175Q0R0 |
| 138 | Tianxue | A250B270C140D200E260F0G390H240I240J190K250L310M295N245O185P175Q195R115 |
| 139 | M1-12×M1-96 | A250B270C215D260E265F380-350G390H240I240J190K250L310M295N245O185P185Q0R115 |
| 140 | Baitangguan | A320-215B270C140D200E265F340G390H235I240J220K290-225L310M295N245O185P175Q195R115 |
| 141 | Tianbao | A250-215B270C215-115D330E265F340G380H240I240J220-190K290-225L310M295N245O185P175Q195-190R115 |
| 142 | Tiangua | A320-250B270C215-115D260E260F350G390H240I240J190K250L310M295N245O185P175Q195R115 |
| 143 | Green Magua | A320B300C140D270E250F380G390H240I240J190K250L350M325N245O185P175Q195R115 |
| 144 | Zhonghua No.1 | A310B420-300C215D305E250F380G390H240I240J190K290L310M295N245O185P175Q195R115 |
| 145 | Fengtian No.3 | A250-215B270C215-115D200E260F350-340G390H240I240J220-190K290-225L310M295N245O185P175Q195R115 |
| 146 | Yinfei No.2 | A215B270C115D200E265F340G390H240I240J220K225L340M295N245O185P175Q195R115 |
| 147 | Qitian No.2 | A320B270C115D200E265F350G390H235I230J190K250L310M295N245O185P175Q190R115 |
| 148 | New Qitian No.1 | A250B270C215D330E265F350G390-380H240I240J190K250L310M295N245O185P185Q0R0 |
| 149 | Longtian No.1 | A320B270C215D200E265F350G380H235I240J190K250L310M295N245O185P175Q195R115 |
| 150 | Xiangxiu No.1 | A0B360-270C140D260-200E265F350G390H240I240J190K250L310M295N245O185P175Q195R115 |
| 151 | Xiangxiu No.4 | A0B270C215D260E260F350G390H240I240J190K250L310M295N245O185P175Q195R115 |
| 152 | Zhenqi | A0B270C215-140D260E265F380-350G390H235I240J190K250L310M295N245O185P175Q195R115 |
| 153 | Aoqi 8111 | A320-250B270C215-115D260E265F380-350G390H240I240J190K250L310M295N245O185P175Q195R115 |
| 154 | Aoqi 819 | A320-215B270C140-115D200E265F350-340G390H240-235I240J220-190K290-225L310M295N245O185P175Q195R115 |
| 155 | Aoqi 8110 | A0B270C215-140D260E260F350G390H240I240J190K250L340-310M325-295N245O185P175Q195-190R115 |
| 156 | Xiangtiancui | A250B270C215-115D260E260F350G390H240I240J220-195K250L310M295N245O185P175Q195-190R115 |
| 157 | Xiangpiaopiao | A250-210B270C215-140D260E260F380-350G390H240I240J190K250L310M295N245O185P175Q195R115 |
| 158 | Huitailang | A215B270C140D330E265F380G380H235I240J190K250L340M325N245O185P175Q190R115 |
| 159 | Tianrumi | A250-210B270C215-140D260E260F380-350G390H240I240J190K250L310M295N245O185P175Q195-190R115 |
| 160 | Jinshuai | A250-210B270C215-140D260E260F380-350G390H240I240J190K250L310M295N245O185P175Q195R115 |
| 161 | Lantiancui | A320B420-270C215D305E250F350G390H240I240J190K250L310M295N245O185P175Q195R115 |
| 162 | Zhentian 2009 | A250B270C215D325-200E265F350G380H240I240J190K250L310M295N245O185P175Q190R115 |
| 163 | Mitianbao | A250B270C215-115D260E265F380-350G390H240I240J190K250L310M295N245O185P175Q195R115 |
| 164 | Xiangtianbao | A250B270C215-115D260E260F350G390H240I240J190K250L310M295N245O185P175Q195R115 |
| 165 | Lantianbao | A305-250B300-270C215D200E260F350G390H240I240J190K250L310M295N245O185P175Q195-190R115 |
| 166 | Zhentianmei | A250B270C215-115D260E260F350G390H240I240J220-195K250L310M295N245O185P175Q195-190R115 |
| 167 | Jingxuan Tiebaqing | A250B270C215D260E260F350G390H240I240J190K250L310M295N245O185P175Q195R115 |
| 168 | Zhonghua Tianbao | A250B270C215-140D260E260F350G390H235I240J190K250L310M295N245O185P175Q195R115 |
| 169 | Shenzhou Tangwang | A250B270C140D0E265F350G390H240I240J190K250L340M325N245O185P175Q190R115 |
| 170 | Zhenshengshi Langua | A305B420-300C115D260E250F380-350G390H240I240J190K290L310M295N245O185P175Q195R115 |
| 171 | Baiyu Tiangua | A250-215B270C215-115D200E265F350-340G380H240I240J220-190K250L310M295N245O185P175Q195R115 |
| 172 | Hefeng New Qitian No.1 | A250B270C215D325E265F350G380H235I240J190K250L310M295N245O185P175Q190R115 |
| 173 | Jingxuan Disease-resistant Hefeng No.5 | A250B270C215D270E260F350-340G390H240I240J190K250L310M295N245O260-185P175Q195-190R115 |
| 174 | Qixin No.3 | A320-215B270C140-115D200E265F350-340G390H235I240J220-190K290-225L310M295N245O185P175Q195R115 |
| 175 | Qixin No.6 | A0B270C140D200E260F350G390-380H235I240J190K250L310M295N245O185P175Q195R115 |
| 176 | Xiangrui No.1 | A0B270C215-140D260E265F350G390H240I240J190K250L340-310M325-295N245O185P175Q195-190R115 |
| 177 | Qitian Wangzi | A250B0C215D260E265F350G390H240I240J190K250L310M295N245O185P175Q195R115 |
| 178 | Xiangyu | A215B270C115D330-200E270F380G390H240I240J220K225L310M295N245O185P175Q195R115 |
| 179 | Longtian No.5 | A320-210B270C140D260-200E260F380-350G390H240I240J220-190K290-225L340M295N245O185P175Q195R115 |
| 180 | Xuewa | A0B270C140-115D260-200E260F380-350G390H235I240J190K250L340-310M295N245O185P175Q195R115 |
| 181 | Longtian No.6 | A250B270C215-140D260E265F350G390H240I240J190K250L340-310M325-295N245O185P175Q195-190R115 |
| 182 | Tianguan No.106 | A215-210B270C140-115D325-200E265F380-340G390H240I240J220-190K290-225L310M325-295N245O185P175Q195-190R115 |
| 183 | Tianguan No.107 | A250-215B270C115D260-200E265F350-340G390H240I240J220-190K290-225L310M295N245O185P175Q195R115 |
| 184 | Tianguan No.109 | A320-250B270C115D325-255E265F380-350G390-380H240I240J220-190K290-225L340-310M295N245O185P175Q195-190R115 |
| 185 | Tianguan No.7 | A320-250B270C145D260-200E265F350G390H240I240J190K250L310M295N245O185P175Q195R115 |
| 186 | Tianguan No.4 | A320-215B270C145-115D260-200E265F380-340G390H240I240J220-190K290-225L310M295N245O185P175Q195R115 |
| 187 | Rikang Q19 | A250B270C140D260E265F350G390H240I240J190K250L340-310M325-275N245O185P175Q195-190R115 |
| 188 | Tiandaoqiu A | A250-215B270C115D200E265F350-340G390-380H240I240J220-190K290-225L310M295N245O185P175Q195-190R115 |
| 189 | Jingmei 009 | A320-250B270C140D260-200E260F350G390H240I240J190K250L310M295N245O185P175Q195R115 |
| 190 | Lvzhou Tianbao | A250B270C215-115D260E260F350G390H240I240J220-195K250L310M295N245O185P175Q195-190R115 |
| 191 | Haotian Tianwang | A250B270C215D260E260F350G390H240I240J190K250L310M295N245O185P175Q0R115 |
| 192 | Super Tianmi | A250B270C215D325E260F350G390H240-235I240J190K250L310M295N245-235O185P175Q195R115 |
| 193 | Baogetian | A320B270C215-115D260E265F350G390H240I240J190K250L310M295N245O185P175Q195R115 |
| 194 | Koukoutian | A320-250B270C215-115D260E260F350G390H240I240J190K250L310M295N245O185P175Q195R115 |
| 195 | Yipin Xianggua | A250B270C140-115D260E265F380-350G390H240I240J190K250L340-310M295N245O185P175Q195R115 |
| 196 | Jilinnongda No.8 | A320-215B270C145D200E250F350-340G390H240I240J190K290-225L310M295N245O185P175Q195R115 |
| 197 | Space Hero | A215B270C140-115D260-200E265F350-340G390H240I240J190K290-225L310M295N245O185P175Q195-190R115 |
| 198 | Xinhuang 2008 | A320-215B270C115D325-200E265F380-340G390H240I240J190K290-225L340-310M325-315N245O185P175Q195R0 |
| 199 | Northeast Guawang | A320-215B270C145D200E250F350-340G390H240I240J190K290-225L310M295N245O185P175Q195R115 |
| 200 | Jilinnongda No.2 | A320-215B270C145D260-200E250F350-340G390H240I240J190K290-225L310M295N245O185P175Q195R115 |
| 201 | Xinfu No.19 | A250B270C215-140D260E260F350G390H240I240J190K250L340-310M315-295N245O185P175Q195-190R115 |
| 202 | Yate | A250B270C215-140D260E260F350G390H240I240J190K250L340-310M315-295N245O185P175Q195-190R115 |
| 203 | Golden Phoenix No.6 | A320-250B270C215-115D255E265F380-350G390-380H240I240J220-190K290-225L340-310M295N245O185P175Q195-190R115 |
| 204 | Yuqingxiang | A250-215B270C215-115D305-200E265F350-340G390-380H240I240J190K290-225L310M295N245O185P175Q195-190R115 |
| 205 | Tiandiaoya | A215B450-270C140-115D200E260F380G380H235I240J190K250L350M295N245O185P175Q195R115 |
| 206 | Hongrangsu | A215B450-280C140D330E260F400G390-380H235I240J190K250L360M295N245O185P175Q195-190R115 |
| 207 | Huapi Sushaogua* | A215B300C105D260E260F400G360H235I240J145K250L310M295N245O185P175Q195R115 |
| 208 | Baipi Sugua | A215B280C140D325E250F380G390H235I230J190K190L310M295N250O185P175Q195R115 |
| 209 | Heipi Sugua* | A215B300C140D330E250F380G395H235I230J145K190L350M295N245O185P175Q195R105 |
| 210 | Huishuzi | A320-215B300-280C140-115D260-200E250F380-340G390-380H235I240J190K250L350-310M295N245O185P175Q195-190R115 |
| 211 | Sweet and crisp | A250-215B270C215D250E265F350G390H240I240J190K250L310M295N245O185P175Q195-190R115 |
| 212 | Guomei | A320-215B270C140-115D200E270F350-340G390H235I240J220-195K290-225L310M295N245O185P175Q195R115 |
| 213 | Gaishi Nonsuch Xiangfei | A320-250B270C215D260E260F350G390H240-235I240J190K250L310M295N245O185P175Q195R115 |
| 214 | Gaishi Nonsuch Prince | A320-215B270C215-115D305-200E260F380-350G390H240-235I240J190K250L310M295N245O185P175Q195R115 |
| 215 | Miaoyu | A320-215B270C115D260-200E265F380-340G390H240I240J220-195K290-225L310M295N245O185P175Q195R115 |
| 216 | Hongxiu | A320-250B270C215-115D260E260F380-350G390H240I240J190K250L310M295N245O185P175Q195R115 |
| 217 | Tianxiang | A250B270C140D260E250F350-340G390H240I240J190K250L340-310M325-295N245O185P175Q195-190R115 |
| 218 | Diamond Tianwang | A215B440-270C115D200E265F340G390H240I240J220K290-225L310M295N245O185P175Q195R115 |
| 219 | Nonsuch Yinsheng | A215B440-270C115D200E265F340G390H240I240J220K225L310M295N245O185P175Q195R115 |
| 220 | Nonsuch Jingxiangyu | A215B0C115D200E265F340G390H240I240J220K225L340M295N245O185P175Q195R115 |
| 221 | Jingmi No.6 | A215B270C115D260-200E265F350-340G390H240I240J190K290-225L310M295N245O185P175Q195-190R115 |
| 222 | Jingmi No.8 | A250B270C215D260E250F350G390H240I240J190K250L310M295N245O185P175Q195R115 |
| 223 | Lingxiu | A250B270C185-140D260E250F350G390-380H235I240J190K250L340-310M315-275N245O185P175Q195-190R115 |
| 224 | Cuigua | A215B280C140D325E250F380G380H240I230J190K250L350M295N245O185P175Q195R105 |
| 225 | Hualei No.2 | A320-215B300-270C140-115D260-200E265F380-340G390H240I240J190K290-225L350-310M325-315N245O185P175Q195R115 |
| 226 | Gagatian | A250B270C215-115D260E260F350G390H240I240J220-195K250L310M295N245O185P175Q195-190R115 |
| 227 | Zhentian No.5 | A320-250B270C215-140D260-200E260F350G390H240I240J190K250L340-310M325-275N245O185P175Q195-190R115 |
| 228 | Jintian 100 | A215B270C115D200E265F340G390H240I240J220-190K225L340-310M295N245O185P175Q195R115 |
| 229 | Nonsuch Yinfeng | A215B270C115D200E265F340G390H240I240J220K225L340M295N245O185P175Q195R115 |
| 230 | Yipin Tianwang | A250B270C215D325E250F350G380H240I240J190K250L310M295N245O185P175Q190R115 |
| 231 | Zetian No.2 | A320-250B270C215-140D260E265F380G390H240I240J220-195K250L340-310M295N245O185P175Q195R115 |
| 232 | Jinfei F_1_ | A250B270C215-140D260E250F350G390H240I240J190K250L340-310M325-295N245O185P175Q195-190R115 |
| 233 | Meiya Huangjindao | A320B300C145D330E250F380G380H235I240J190K250L310M295N245O185P175Q195R115 |
| 234 | Gaochun Gantian No.1 | A320-250B270C215-140D260-200E260F350G390H240I240J190K250L310M295N245O185P175Q195R115 |
| 235 | Gaochun Rainbow No.7 F_1_ | A320-250B270C140D260-200E260F350G390H240I240J190K250L310M295N245O185P175Q195R115 |
| 236 | Longqing No.1 | A250-215B270C215-115D255E260F350G390-380H240I240J220-190K290-225L310M295N245O185P175Q190R115 |
| 237 | Longqing No.2 | A320-215B270C145-115D200E260F350-340G390H240I240J220-190K290-225L310M295N245O185P175Q195R115 |
| 238 | Longqing No.3 | A215B340-270C115D250-200E260-205F370-340G420-390H270-240I250-240J245K225L310M345-295N245-235O280-185P185-175Q195-170R180-115 |
| 239 | Longqing No.4 | A320-215B270C145-115D260-200E260F380-340G390H240I240J220-190K290-225L310M295N245O185P175Q195R115 |
| 240 | Qitian No.1 Tiangua | A250B270C215D325E260F350G380H240I240J190K250L310M295N245O185P175Q190R115 |
| 241 | Qitian No.2 Tiangua | A320B270C115D200E260F350G390H235I230J195K250L310M295N245O185P175Q190R115 |
| 242 | Qitian No.3 Tiangua | A320B270C215-115D270E260F350G390H235I240J190K250L310M325N0O0P175Q190R115 |
| 243 | Fuer No.1 Tiangua | A250-215B270C215D270E250F350G390H240I240J190K250L310M295N245O185P175Q195R115 |
| 244 | Fuer No.2 Tiangua | A320-250B270C215-115D2E250F350-340G390-380H240I240-230J220-190K250L340-310M295N245O185P175Q195-190R115 |
| 245 | Yongtian No.3 Tiangua | A250-215B270C215-115D305-200E260F350-340G390-380H240I240J220-190K290-225L310M295N245O185P175Q195-190R115 |
| 246 | Yongtian No.9 Tiangua | A320-250B270C115D255E260F380-350G390-380H240I240J220-190K290-225L340-310M295N245O185P175Q195-190R115 |
| 247 | Zetian No.1 Tiangua | A320-215B270C140D255-200E260F380-350G390H240I240J190K290-225L310M295N245O185P175Q195-190R115 |
| 248 | Meiya Futian No.1 Tiangua | A320-215B270C140-115D200E260F350-340G390H240I240J220-190K290-225L340-310M295N245O185P175Q195R115 |
| 249 | Qi 2007-1 Tiangua | A320B270C115D255E265F390G390H235I240J220-190K290-225L340-310M295N245O185P175Q195R115 |
| 250 | Qi 05-5 Tiangua | A320-215B270C115D270-200E265F390-340G390H235I240J220-190K290-225L310M295N245O185P175Q195R115 |
| 251 | Longqing ZT-8 Tiangua | A250-215B270C215-115D270-200E265F350-340G390-380H235I240J220-190K290-225L310M295N245O185P175Q195-190R115 |
| 252 | Longqing Qiutian Tiangua | A250-215B270C175-115D255-200E270-205F380-340G390H360-235I275-240J220K225L340-310M345-295N245O260-185P190-175Q195-170R180-115 |
| 253 | Qi Xiangmi Tiangua | A250-215B270C140-115D270-200E260F350-340G390-380H235I240J190K290-225L340-310M295N245O185P175Q0R0 |
| 254 | Qitian Cuitiangua | A250-215B270C115D270-200E260F350-340G390H235I240J220-190K290-225L310M295N245O185P175Q195R115 |
| 255 | Cuiyu | A250-215B270C115D270-200E260F350-340G390-380H235I240J220-190K290-225L310M295N245O185P175Q195-190R115 |
| 256 | 16H | A185B340C185D255E270F370G430H265I250J245K225L320M345N260O220P205Q190R180 |
| 257 | WI998* | A185B350C170D255E205F370G430H270I250J250K225L320M345N260O220P185Q195R150 |
| 258 | 16A | A185B340C185D255E270F370G430H265I250J245K225L320M345N260O220P205Q190R150 |
| 259 | Elizabeth Male Parent* | A250B340C185D250E205F370G440H275I275J245K225L310M345N235O260P185Q170R170 |
| 260 | TN | A185B350C185D255E205F360G430H235I275J245K210L320M325N260O220P200Q170R180 |
| 261 | BF | A215B0C185D255E205F370G430H265I250J245K225L310M345N235O280P185Q170R115 |
| 262 | Yuan H3 | A250B340C185D255E205F370G390H270I250J190K180L310M345N235O260P185Q195R115 |
| 263 | S3 | A185B270C185D255E205F370G440H240I240J245K225L310M295N260O185P185Q170R180 |
| 264 | M-135 | A180B350C145D255E205F370G360H265I250J250K225L310M325N235O280P190Q170R150 |
| 265 | M-012 | A250B270C185D255E205F370G390H265I275J245K295L310M345N290O260P200Q170R180 |
| 266 | M-008 | A250B0C115D255E205F370G390H265I230J245K225L320M300N260O185P180Q170R115 |
| 267 | Changes 3 | A185B350C185D255E205F370G440H265I240J245K180L310M345N260O220P185Q195R180 |
| 268 | Niusi149 | A185B350C175D255E205F370G360H235I250J245K180L310M345N290O280P185Q170R180 |
| 269 | Tiedanzi* | A215B340C175D250E205F370G360H265I250J245K180L310M345N315O280P185Q170R150 |
| 270 | Hetao Migua | A215B0C170D250E205F370G360H265I250J245K225L310M345N300O280P185Q170R150 |
| 271 | Bailangua* | A185-180B340-270C185D255E205F370G430H275I275J245-220K225L310M345N290-235O220P195Q170R180-150 |
| 272 | Hongxincui* | A180B350C180D250E325F360G430H265I250J250K210L320M325N235O220P205Q170R150 |
| 273 | Kalakesai* | A185B0C180D250E325F370G420H270I275J250K225L320M325N235O220P205Q170R150 |
| 274 | PMR45* | A225B340C170D250E205F370G360H235I250J220K225L320M345N260O265P190Q195R150 |
| 275 | Jiuyimaowakasi | A215B350C175-125D255E250-205F370G440H265I250J245K210L310M315-295N260O280P185Q190R150 |
| 276 | Cinderella | A215B350C175D255E205F370-360G440H360-235I250J245K210L310M345-295N235O280P185Q170R150 |
| 277 | Yourangsika* | A215B350C175D255E205F370G360H270I250J250K210L310M345N260O280P185Q170R150 |
| 278 | Xiaoguan | A180B340C175D250E205F370G360H265I250J250-245K180L310M345N235O280P185Q170R150 |
| 279 | Yikawa | A215B350C175D255E205F370G360H235I250J245K180L310M345N260O280P185Q170R150 |
| 280 | Gulumie | A240-215B350C175D255E205F370-360G440-430H235I250J245K210L310M0N260O280P185Q170R150 |
| 281 | Rio Gold | A185B340C175D255E250-205F370-360G440H360-240I250J250K180L320-310M345N260O260P180Q170R150 |
| 282 | Pezsiano | A215B340C175D255E205F370G360H265I240J245K210L310M345N260O275P185Q170R150 |
| 283 | Peslita | A225B340C175D255E250F370G440H265I275J245K225L310M345N235O270P205-180Q190R180 |
| 284 | Golden Pheonix (Yin) | A185B340C180D255E325-205F360G440-430H360-235I250J245K210L320M325N235O220P205-180Q170R180 |
| 285 | Midu | A215B0C115D260-200E265-205F370-340G430-380H360-235I250-240J245K225L310M0N0O0P185Q0R0 |
| 286 | Green Angel | A215-185B340-270C180-115D255-200E265-205F370-340G430-380H360-235I250-240J245K225L310M345-295N235O275-185P205-175Q195-170R115 |
| 287 | Guifei F_1_ | A250-185B270C180D255E205F370G430-380H270I275J245K295-225L310M345N290-245O280-260P205-175Q170R180 |
| 288 | Dingtian No.3 | A215B350-270C170-115D260-200E265-205F370-340G430-380H360-235I240-230J245K225L320-310M300-295N245O275-185P185-175Q195R180-115 |
| 289 | Fenglei | A250-215B340-270C170D255E205F360G430-380H235I275-250J245K225-210L320-310M345-325N260O0P175Q195-190R115 |
| 290 | Nonsuch Huangyu | A250-215B270C170D255E265-205F360G430-380H235I240-230J245K290-225L320-310M325-300N245O185P205-185Q195-170R180-115 |
| 291 | Luhoutian No.1 | A225-185B340C180-115D255E205F370G430H265I275-250J245-220K250-225L320-310M345-300N235O250P180Q170R180-150 |
| 292 | Lucky-52 | A185B340-270C180D0E325-205F370-360G430H360-235I250J250K250-210L320M325-275N235O220P200Q170R180-115 |
| 293 | Lucky-8 | A210-185B340C180D255E205F370G440-430H265I250J250K225L320-310M325-295N235O220P190Q170R150-115 |
| 294 | Zhongtian No.1 | A305-250B270C180D255E260-205F360G430-380H240I240-230J245K290-225L310M325-300N245O185P175Q170R180-115 |
| 295 | Zhongtian No.2 | A210-185B340-270C180D260E205F380-360G430H265I250J245K225L320-310M345-325N260O275P190Q170R180 |
| 296 | Network Era | A305-250B340C180D260E205F370G430-360H265I275-240J245-220K250-225L310M345-300N245O270P200Q170R180-160 |
| 297 | Xuehong | A305-250B270C180D255E260-205F370G440-380H0I275-250J245K225L310M345-325N290O260P205-185Q170R180 |
| 298 | Yongtian No.3 | A305-250B340-280C180D255E205F370-360G420-380H265I275-250J245K250-225L335-310M325-300N260O220-185P185Q170R150-115 |
| 299 | Yongtian No.5 | A305-210B350-280C180D255E205F360G430-420H265I275J245K210L335-320M325N260O220P185Q170R180-150 |
| 300 | Zhetian No.2 | A305-250B350C170D270E205F370G360H270I240-230J245-220K250-200L320-310M300N235O260P180Q170R160-150 |
| 301 | Zhetian No.3 | A305-250B350C170-115D270E205F370G430-360H270I240-230J245-220K250-200L320-310M300N235O260-190P180Q170R160-150 |
| 302 | Fengtian No.1 | A305-215B270C170D255E260-205F360G430-380H235I275-230J245K290-225L320-310M325-275N245O185P205-175Q170R180-115 |
| 303 | Fengtian No.3 | A305-250B270C115D270-200E260F340G380H235I240J190K290-225L310M295N245O185P175Q195R115 |
| 304 | Fengtian No.8 | A305-250B350C180D255E205F370G430-380H265I230J245K225L320-310M300N235O220-185P205-180Q170R180-160 |
| 305 | Jinmi | A210-180B340C185-140D270-255E205F370-360G440-430H265I250J245-220K295-225L310M345-325N260O260P180Q170R180-115 |
| 306 | Jinrui | A210-180B340C185-140D270-255E205F370-360G440-430H265I250J245-220K295-225L310M345-325N260O260P180Q170R180-115 |
| 307 | Jinli | A250-220B270C175D255E260-205F360G380-360H235I275-230J245-190K290-225L310M325-300N235O185P185-175Q170R115 |
| 308 | Yuxiang | A215-185B270C185D255E205F370G430-380H265I275J245-190K295-225L310M345N290O260P205-175Q170R170 |
| 309 | Huanghemi No.6 | A305-215B340-270C185D255E205F370G430H265I250J220K225L310M345N290O270P200Q170R170 |
| 310 | Huanghemi No.3 | A215-185B340-270C185D255E205F370G360H265I250J220K225L310M345N290O275P200Q170R170 |
| 311 | Gantian Yulu | A250-220B340-270C140-105D255E205F370G430H265I275-250J245-220K225L310M345-300N260O250P185Q170R170 |
| 312 | Jinhui No.1 | A215-185B340C170D255E205F360G440-430H265I275-250J250K225L310M325N235O220P185Q190-170R170 |
| 313 | Red Pearl Net | A305-250B350C175D270-255E205F370G360H265I230J245K225L320M345-300N235O275P185Q170R150 |
| 314 | Pearl Net | A305-250B340-270C185D270E265-205F370G440-360H265I230J245-220K225L320M345-300N235O260P185Q170R150 |
| 315 | Sutian No.1 | A225-220B440C180D270-255E260-205F370G380H235I275-250J245K225L310M345N235O260-250P185Q170R170 |
| 316 | Sutian No.2 | A225B440C180D270-255E260-205F370G380H235I275-250J245K225L310M345N290O250P185-180Q0R0 |
| 317 | Fengwei No.4 | A215-185B350-270C185D255E260-205F350G440H235I230J250-245K225L320M345-295N235O280-185P190-175Q170R150 |
| 318 | Xianguo | A215-185B340C180D255E205F350-340G440H265I250J250K250-225L320M325N235O220-185P205Q170R150 |
| 319 | Huangpi 9818 | A305-250B350-270C180D255E205F350G430H235I250-230J250K250-225L320M325-300N235O220P185Q170R180-160 |
| 320 | Lvpi 9818 | A305-250B350-340C185D270-255E205F350G430-360H265I230J250-245K225L320M300N235O220P190Q170R150 |
| 321 | Jinli No.2 (Little Jinli) | A305-215B340-270C170-140D270-255E265-205F350G390H265I250-230J245K290-225L350-310M345-300N245O250P180Q195-170R180-115 |
| 322 | Xuelihong | A215-185B350-280C180D250E205F350G430-420H360-235I275J250-245K210L335-320M325N260O220P185Q170R180-160 |
| 323 | Zaoxianzui | A305-185B340C180D250E205F350G440-360H360-235I250-240J250-245K250-210L320-310M325N235O220-185P205-180Q195-170R150-115 |
| 324 | Xinmi Hybridization No.9 (Huangzuixian) | A250-215B340C175D250E205F370-350G440-360H265I250J250-245K180L320-310M345-325N260O280-220P190Q170R150 |
| 325 | Xinmi No.36 (Golden Dragon) | A215-185B340-270C170D250E205F370-350G440-430H360-235I250J250K250-210L320-310M325N235O220P185Q170R180-160 |
| 326 | Xinji Xuelian (Xinmi No.21) | A215-180B340C185-115D250E265-205F370-350G430-390H360-235I250-240J245K225L310M345N235O280-185P180Q170R180-115 |
| 327 | Xueli | A215-185B340-270C185D250E205F350G430-390H360-235I275J245K250-210L320-310M325-300N260O220P185Q170R180 |
| 328 | Golden Pheonix (Xinmi No.28) | A215-185B340-270C185D250E270-205F350G440-430H360-235I250J250K210L320M325N235O220P205-185Q170R180 |
| 329 | Xinhongxincui (Xinmi No.24) | A215-185B340C180D250E205F350G430H360-235I250J250-245K210L320M325N260-235O220P205-185Q170R180 |
| 330 | Emerald | A305-215B350-270C185D270E205F370-350G0H265I230J245K225L320M300N235O280P205-185Q170R150 |
| 331 | Jinli No.1 (Big Jinli) | A215-185B340C175-140D250E205F350G390H270I250-230J245K290-225L350-310M345-300N245O250-185P190Q195-170R180-115 |
| 332 | F11 | A305-250B340C170D270-255E205F370G430-360H360-235I230J245K225L320M345-300N235O220P205Q170R150 |
| 333 | F09-1 | A305-250B270C185-115D250E205F370G430-390H265I275-250J245K290-225L310M345-300N260O280-260P190Q170R180 |
| 334 | Play Melon* | A250B370C115D0E270F380G380H235I230J145K250L350M295N245O185P175Q195R115 |
| 335 | Red Fruit (wild) | A320B280C100D0E195F380G275H360I275J140K290L310M295N245O340P175Q195R115 |
| 336 | Little Melon (wild)* | A215B400C145D325E250F390G390H235I230J145K250L350M295N245O185P175Q190R115 |
| 337 | Qingpi Caigua No.1* | A225B280C145D330-200E250F400G390H235I230J145K290L310M295N245O185P175Q195R115 |
| 338 | Heipi Caigua | A250-215B300-270C140D200E250F380G390H235I230J190K250-190L350M295N245O185P175Q195R105 |
| 339 | Yutian No.1 | A350-320B270C140D200E265F350G390H235I240J220-195K250L310M295N245O185P175Q195R115 |
| 340 | Tianmeiwuxian | A305-250B270C215-115D200E260F340G390H235I240J220-195K290-225L310M295N245O185P175Q195R115 |
| 341 | No.19 | A250B270C185D250E250F350G390H240I275J190K225L310M345N245O260P190Q190R150 |
| 342 | 8-2-3-1 | A350B270C140D330E265F350G390H240I240J190K250L340M295N245O185P175Q195R115 |
| 343 | Gaotang Yucui | A250B420-270C0D260E260F350G390H240I240J190K250L310M295N245O185P175Q195R115 |
| 344 | Teda Shengkaihua | A215B280C115D200E250F340G390H235I240J190K250L350M295N245O185P175Q190R115 |
| 345 | Jinmi Tianshuai | A305-250B270C215D260E260F350G0H240I240J190K250L310M295N245O185P175Q195R115 |
| 346 | Jindaowang | A350-320B300C145D2E250F370G395H225I240J195K250L310M295N245O185P175Q195R115 |
| 347 | Big Longtian No.1 | A320B270C115D260E265F350G395H235I240J195K250L310M295N245O185P175Q195R115 |
| 348 | Jingpi Lvmagua | A350-215B300C140D260E250F370-340G395H240I240J190K250L350-340M325N245O185P175Q195-190R115 |
| 349 | Jindaozi | A250-215B300C140D325E250F340G395H235I240J195K250L310M295N245O185P175Q195R0 |
| 350 | Jingpin Xuemeiren | A320B270C140D200E265F350G395H235I240J195K250L310M295N245O185P175Q195R115 |
| 351 | Balengcui | A320B270C140D200E265F350G395H235I240J195K250L310M295N245O185P175Q195R115 |
| 352 | Hongrangsu | A250-215B280C140D330E260F400G390H235I230J190K250L360M295N245O185P175Q195R115 |
| 353 | Qitian No.8 | A305-250B270C215D260E260F350G395H240I240J190K250L310M295N245O185P175Q195R115 |
| 354 | Huapimian | A250-215B270C115D330E250F370G390H240I240J190K250L350M295N245O185P175Q190R115 |
| 355 | Jingpin Balixiang | A310B350-270C140-115D2E250F340G390H235I240J195K250L310M295N245O185P175Q195R115 |
| 356 | Tiancuiang | A240-210B270C140D260E265F370G395H240I240J195K250L310M295N245O185P175Q195R115 |
| 357 | Big Hongchengcui | A320B270C115D260E265F350G395H235I240J195K250L310M295N245O185P175Q195R115 |
| 358 | Improved Pohongpi | A320-215B300-270C140-115D200E250F350G395-390H235I0J195K250L310M295N245O185P175Q195-190R115 |
| 359 | Shengkaihua | A215B270C140-115D200E250F370G390H235I240J190K250L310M295N245O185P175Q195R115 |
| 360 | Improved Huangjindao | A350-320B270C115D2E265F350G395H235I240J190K250L310M325N245O185P175Q195R0 |
| 361 | Big Huishuzi | A250-215B280C115D200E250F340G390H235I240J190K250L350M295N245O185P175Q190R115 |
| 362 | Qitian No.1 | A250B270C215D325E260-200F350G380-350H240I240J190K250L310M295N245O185P175Q190R115 |
| 363 | Big Balixiang | A310B350-270C115D325E250F370-340G390H240I240J195K250L310M295N245O185P175Q195R115 |
| 364 | Shidaogou | A350215B270C115D330-270E260F370-350G395H240I240J190K250L310M325-315N245O185P175Q190R115 |
| 365 | Baiyu No.1 | A215B280C115D260E250F370G390H240I240J190K250L340M0N245O185P175Q195R115 |
| 366 | Zetian No.5 | A350-320B0C140D260E265F370G395H240I240J195K250L310M295N245O185P175Q195R115 |
| 367 | Dongtian 002 | A305-250B270C215-115D325-200E265F350-340G390H240I240J190K250L310M295N245O185P175Q195-190R115 |
| 368 | TopMark* | A225B340C170D250E205F370G360H235I250J245K225L320M345N260O220P205Q190R150 |
| 369 | T6 | A250B340C175D250E205F360G360H265I250J245K225L310M345N260O250P180Q170R180 |
| 370 | Fengtian | A215-185B340-270C175-115D250E265-205F360G430-395H240I250-230J245-190K290-225L310M300-295N245O250-185P185-175Q170R0 |
| 371 | Kang 2 | A215B350C170D260E205F370G395H265I240J250K190L310M300N235O280P195Q190R160 |
| 372 | BT3 | A350-185B340-300C175-145D250E270-205F360G430-395H360-265I250-240J245K290-225L310M300-295N260-245O250-185P185-175Q170R105 |
| 373 | M-074 | A215B300C175D255E205F370G430H240I250J245K225L320M345N0O280P195Q170R105 |
| 374 | M-130 | A350-215B340-270C175-115D260E270-205F350G395H235I250J245K295-210L320M345N260-245O250-185P185-175Q190R115 |
| 375 | M-021 | A250B340C185D250E205F370G440H265I275J245K225L310M345N235O260P185Q170R150 |
| 376 | Annong No.3 | A215B340C175D260E270-205F360G430-395H360-235I230J245K210L320M345-300N260-235O250-185P185Q190-170R115 |
| 377 | MRIL7-32 | A225B0C185D255E270F370G430H265I250J220K225L320M345N260O220P205Q190R180 |
| 378 | MRIL7-35 | A185B340C185D250E205F370G430H235I250J245K225L320M345N250O220P205Q0R180 |
| 379 | MRIL7-47 | A225B440C170D255E270F370G360H235I250J245K180L320M345N250O220P205Q190R150 |
| 380 | MRIL7-50 | A225B0C170D255E205F370G360H265I250J220K180L320M345N250O220P205Q190R150 |
| 381 | MRIL7-51 | A185B0C170D250E205F370G360H265I250J220K180L310M345N250O220P205Q190R180 |
| 382 | MRIL7-64 | A225B340C185D255E270F370G360H265I250J245K180L310M300N260O220P205Q190R180 |
| 383 | MRIL7-87 | A225B440C170D250E205F370G360H265I250J220K180L310M345N250O220P205Q190R180 |
| 384 | MRIL7-142 | A225B340C185D255E270F370G360H265I250J220K225L310M300N260O220P205Q190R150 |
| 385 | MRIL7-153 | A185B440C170D250E205F370G360H235I250J245K180L310M345N260O220P205Q190R180 |
| 386 | MRIL7-165 | A185B340C185D255E270F370G360H235I250J220K180L310M345N250O220P205Q190R180 |
| 387 | PI614526 | A215B255C185D215E210F360G420H225I245J190K210L350M325N235O190P180Q175R110 |
| 388 | PI614572 | A250B270C140D270E250F360G390H240I240J195K250L300M300N245O185P175Q190R115 |
| 389 | PI614433 | A185B0C125D305E375F390G430H235I275J190K290L300M310N235O185P170Q175R100 |
| 390 | PI614281 | A240B440C130D330E250F360G360H0I275J250K290L350M310N235O190P185Q175R115 |
| 391 | Meishizhe | A215B340C175D250E205F370G440-390H265I250J245K210-180L300M345N235O280P185Q170R150 |
| 392 | Xiaxi 43 | A250B350-270C185D270E265-205F370G360H265I230J245K225L310M300N235O260P0Q170R150 |
| 393 | Xiaxi 44 | A250B350-270C185D270E265-205F370G360H265I230J245K225L310M300N235O260P180Q170R150 |
| 394 | Xiaxi 45 | A250B350C185-115D270E205F370G360H270I230J245K225L310M300N235O260P180Q170R150 |
| 395 | Xiaxi 47 | A250B350C185-115D270E205F370G440H265I250-230J245K250-200L310M300N235O280-190P185Q170R150 |
| 396 | Xiaxi 50 | A225-215B340C175-140D240E205F360G390H235I250-230J0K210L310-300M345-300N260O250P0Q170R0 |
| 397 | Xiaxi 51 | A250-225B350-340C175D240E265F370G440H270-235I250-230J245K225L320M345-300N260-235O190P180Q190-170R150-115 |
| 398 | Xiaxi 52 | A250-225B350-340C185-175D250E265-205F370G440H270-235I250-230J245K225L320M345N235O190P185Q170R150 |
| 399 | Xiaxi 54 | A250-185B340-270C180-175D250E205F370G440H265I275J220K225L300M345N235O280P200Q170R180 |
| 400 | Xiaxi 55 | A225-185B350-270C185D250E260-205F370-360G440H240I275J245-220K225L300M345-325N290O280-250P175Q170R180-115 |
| 401 | Xiaxi 57 | A185B350C180-170D250E205F370-360G440H265I250J245-220K225L300M345N235O250P185Q170R180 |
| 402 | Xiaxi 58 | A250-185B340-270C185-140D270-250E400-260F370-360G440-390H270I250-230J220K290-225L300M325-315N245O250-185P175Q195-170R180-115 |
| 403 | Xiaxi 61 | A250-220B350C175-115D270E205F370G440-360H275I240-230J245-220K250-200L320-300M300N235O280-260P180Q190-170R160-150 |
| 404 | Xiaxi 63 | A250B350C185-115D270E265-205F370G360H275I230J245K225L320M300N235O260P180Q170R150 |
| 405 | Xiaxi 71 | A215-185B340C175-140D270-250E205F370G390H275I240-230J245-220K290-200L310M345-300N235O280P185Q190-170R160-150 |
| 406 | Xiaxi 72 | A250-225B340C185-175D270E265-205F370G360H270I240-230J245-220K250-200L320-310M0N235O280-260P180Q190-170R150 |
| 407 | Xiaxi 73 | A215-185B340C175-110D270-255E205F370G430H270I250-240J245-220K250-200L310M300N235O280P185Q190-170R180-160 |
| 408 | Xiaxi 74 | A250B350C175-115D270-250E205F370G430-360H270I230J245K225L320M300N260-235O260-185P180Q195-170R150 |
| 409 | Xiaxi 75 | A215B340C175D270-250E205F370-360G390H270-235I250-240J245-220K210-200L310M345-300N260-245O280-250P180Q190-170R160-115 |
| 410 | Xiaxi 76 | A250B350-340C185-115D270E265-205F370G360H270I230J245K225L320M300N235O260P180Q170R150 |
| 411 | Xiaxi 77 | A250B350-340C185D270E265-205F370G360H270I230J245K225L320M300N235O260P180Q170R150 |
| 412 | Xiaxi 78 | A250B350C185D270E265-205F370G360H270I230J245K225L320M300N235O260P180Q170R150 |
| 413 | Xiaxi 79 | A250B350C185-115D270E265-205F370G360H270I230J245K225L320M300N235O260P180Q170R150 |
| 414 | Xiaxi 81 | A250-215B340-270C175-115D250-200E265-205F370-340G390H240I250-240J245K225L320-310M345-295N260-245O220-185P175Q195-190R150-115 |
| 415 | Xiaxi 82 | A250-215B340C175-115D250-200E265-205F370-340G390H240I240-230J245K225L320-310M345-295N260-245O220-185P175Q195-190R150-115 |
| 416 | Xiaxi 83 | A215B340-270C175-115D250-200E265-205F370-340G390H240I240-230J245K225L320-310M345-295N260-245O220-185P175Q195-190R150-115 |
| 417 | Xiaxi 84 | A250-215B340-270C175-115D250-200E265-205F370-340G390H240I240-230J245K225L320M345N260O220P185Q195-190R150-115 |
| 418 | Xiaxi 85 | A215B270C115D200E260F340G380H240I230J190K290L310M295N245O185P175Q195R115 |
| 419 | Xiaxi 86 | A250B270C115D200E265F340G390H240I240J220K225L310M295N245O185P175Q195R115 |
| 420 | Xiaxi 87 | A250B340C180D255E205F360G430H270I230J220K225L300M345N260O220P175Q195R150 |
| 421 | Xiaxi 88 | A250-225B340-270C180-175D260-250E205F370-360G440H270I250-230J245K225L320M345-300N260-235O220P180Q190-170R150 |
| 422 | Xiaxi 89 | A215B350C175D260E205F370G440-360H270I250-240J250-220K200L310M300N235O280P180Q190-170R150-115 |
| 423 | Xiaxi 90 | A250-225B350C175-115D260-250E205F370G360H270-240I250-230J245K225L320M345-300N260-235O220P185Q190-170R150 |
| 424 | Xiaxi 91 | A225B350C175D260-255E265-205F370G440-360H270-240I250J245K250-225L320M345N260O220P180Q190-170R160 |
| 425 | Xiaxi 92 | A250-225B340C185D260-250E205F370G440-360H270I250-230J245K250-200L320M345-300N245O190P185Q190-170R150-115 |
| 426 | Xiaxi 93 | A225-215B340C175D250-240E205F360G440-390H270-240I250J250-220K210L310M345N260-235O250P185Q190R180-115 |
| 427 | Xiaxi 94 | A215-185B340C175-145D240E205F370-360G440-390H270I230J250-220K290-210L320-310M345N260-235O250P185Q190-170R150-115 |
| 428 | Xiaxi 95 | A225-215B340C175-145D250E265-205F370-360G440-360H240I250-230J245-220K250-210L320M345N260O220P180Q190-170R150 |
| 429 | Xiaxi 96 | A250-185B340-270C185-115D255-200E265-205F360-340G390H270I275-230J245-220K225L310M325-295N235O250P200-175Q195R180-115 |
| 430 | Xiaxi 97 | A225-185B340-270C185D250E205F370-360G390H270I275J220K225L310M345-325N260O250-220P195Q170R180 |
| 431 | Xiaxi 98 | A250-225B350-340C185-175D260-250E205F370G360H270I250-230J245K225L320M345-300N290-235O280P200-185Q190-170R150 |
| 432 | Xiaxi 99 | A250-225B350-340C185-175D260-250E205F370G360H270-240I250-230J245-220K225L320M345-300N260-235O220P185Q190-170R150 |
| 433 | Xiaxi 100 | A250-225B350-340C170-115D260-250E205F370G360H270-240I250-230J245-220K225L320M345-300N260-235O220P185Q190-170R150 |
| 434 | Xiaxi 101 | A215-185B340-270C175D250E205F360G390H270-235I250-230J245K250-210L310M345-325N260O250P185Q190-170R180-115 |
| 435 | Xiaxi 102 | A185B350C180D250E205F370-360G390H270I250J245-220K225L310M345-325N235O250P185Q170R180 |
| 436 | Xiaxi 103 | A185B350-340C180D250E205F370-360G390H270I250J245-220K250-210L310M345-325N235O250P185Q170R180 |
| 437 | Xiaxi 104 | A185B350-340C180D250E205F370-360G380H270I250J245-220K250-225L310M345-325N235O250P185Q170R180 |
| 438 | Xiaxi 105 | A185B340-270C185-145D255E205F370-360G390H270I275-250J220K250-225L335-310M345-325N235O250-220P180Q170R180-150 |
| 439 | Xiaxi 106 | A185B340C180D250E400-205F370-360G390H270I250J245-220K225L310M345-325N290O250P185Q170R180 |
| 440 | Xiaxi 107 | A185-180B350-270C170D250E400-205F370-360G0H270I250J245-220K225L310M345N235O250P185Q170R180 |
| 441 | Xiaxi 108 | A185-180B350-270C185D250E205F370G440-430H270I250J250-220K225L310M345-325N245O250P205-175Q170R180-115 |
| 442 | Xiaxi 109 | A250B340-270C170D250E205F370G430H270I250-230J245K225L320M345N260O220P185Q190R150 |
| 443 | Xiaxi 110 | A225B340C185D250-240E205F370-360G430-360H270I250-245J245K225L320M345-300N260O220P205-185Q190R150-115 |
| 444 | Yinmi No.3 (Yin) | A215-185B340-270C180-115D250E205F370-350G440-430H270I250J245K225L310M325-300N260O250P185Q170R180 |
| 445 | Russia No.2 (Yin) | A185B340-270C185D255E205F370G430H270I275J220K225L310M345N290O275P200Q170R180 |
| 446 | Minitian No.1 | A250-185B340C170-140D250E260-205F370-350G430-390H275I250-230J250-220K250-210L320M345N260O250-220P185Q195-190R180-150 |
| 447 | Minitian No.2 | A250-185B340C170-140D260-250E260-205F370-350G390-360H275I250-230J250-220K210-200L320M345N260O250P185Q190-170R180-150 |
| 448 | Minitian No.3 | A225-185B340C170-140D260-250E260-205F370-350G390-360H275I250-230J250-220K210-200L320M345N260O250-220P185Q190-170R180-150 |
| 449 | Minitian No.4 | A225-215B340C170-140D250E205F350G395-390H235I250J250-220K250-210L310M345N260O275P185Q190-170R180 |
| 450 | Huangjinbao (Yin) | A185B340-270C185D255E205F370G360H275I250J220K225L310M345N290O280P200Q170R180 |
| 451 | Qingpi Caigua 2 | A215B300C145D2E250F400G390-380H235I230J190K250L360-310M295N245O185P175Q195R105 |
| 452 | Qinglong Caigua | A215B300C140D325E250F400G390H240I230J190K250L360M295N245O185P175Q195R110 |
| 453 | Sucui Baicaigua | A225B280C145D270E250F400G390H240I230J190K190L350M295N250O185P175Q195R105 |
| 454 | Huapi Caigua | A215B280C160D325-260E250F400G390H240I230J190K250L350M295N235O220-185P175Q195R110 |
| 455 | Qinghuapi Hongrang Caigua | A215B360-280C140D270E260F400G0H240I240J190K290L310M295N235O185P175Q195R115 |
| 456 | IranH | A180B0C185D250E205F360G420H270I250J190K225L310M325N290O220P190Q170R150 |
| 457 | Topmark | A180B360C185D250E205F360G420H270I250J190K225L310M325N290O220P190Q170R150 |
| 458 | Vedrantais | A225B340C175D250E205F370G430H270I250J245K225L320M300N250O220P185Q190R115 |
| 459 | PMR5 | A225B340C170D240E205F380G360H240I250J245K225L320M345N250O220P205Q190R115 |
| 460 | Edisto47 | A215B0C180D255E265F380G360H275I250J245K225L320M345N230O275P185Q170R150 |
| 461 | PI414723 | A215B255C160D255E325F390G390H245I275J160K250L360M315N230O220P190Q170R115 |
| 462 | MR-1 | A215B0C175D250E325F350G360H275I275J145K210L310M330N245O220P200Q170R150 |
| 463 | PI124111 | A185B340C215D250E325F380G360H275I250J245K225L310M345N245O220P200Q175R150 |
| 464 | PI124112 | A215B400C105D260E325F360G420H270I275J245K165L310M330N230O205P200Q170R180 |
| 465 | PMR6 | A225B340C170D250E205F380G360H240I250J245K225L320M345N250O220P205Q190R150 |
| 466 | Nantais | A250B340C170D250E205F380G430H275I250J245K225L320M345N250O220P185Q190R115 |
| 467 | 185 | A215-185B340C180D250E205F360G390H240I275-250J250-220K250-210L320-310M345N250-230O275P175Q195-170R180 |
| 468 | 150 | A215-185B340C180D250E205F360G430-390H240I250J250-220K250-210L320-310M345N250-230O275P175Q195-170R180 |
| 469 | 192 | A250-185B340-270C175D250E205F360G430-390H240I250J245-220K250-210L320-310M345N250-230O275P175Q195-170R180-115 |
| 470 | 117 | A185B340C180-115D250E265-205F360G440-390H245I275-250J245K225L310M345-325N290-230O280-260P190Q170R180-115 |
| 471 | Ningnongtian No.3 | A250B270C180D250E205F380G430-390H245I250-240J245K290-225L310M345-295N290-230O250-185P175Q195-170R115 |
